# With a little help from my friends: the roles of microbial symbionts in insect populations and communities

**DOI:** 10.1098/rstb.2023.0122

**Published:** 2024-06-24

**Authors:** Piotr Łukasik, Michał R. Kolasa

**Affiliations:** Institute of Environmental Sciences, Faculty of Biology, Jagiellonian University, 30-387 Krakow, Poland

**Keywords:** microbiome, symbiosis, facultative endosymbiont, adaptation, *Wolbachia*, barcoding

## Abstract

To understand insect abundance, distribution and dynamics, we need to understand the relevant drivers of their populations and communities. While microbial symbionts are known to strongly affect many aspects of insect biology, we lack data on their effects on populations or community processes, or on insects' evolutionary responses at different timescales. How these effects change as the anthropogenic effects on ecosystems intensify is an area of intense research. Recent developments in sequencing and bioinformatics permit cost-effective microbial diversity surveys, tracking symbiont transmission, and identification of functions across insect populations and multi-species communities. In this review, we explore how different functional categories of symbionts can influence insect life-history traits, how these effects could affect insect populations and their interactions with other species, and how they may affect processes and patterns at the level of entire communities. We argue that insect-associated microbes should be considered important drivers of insect response and adaptation to environmental challenges and opportunities. We also outline the emerging approaches for surveying and characterizing insect-associated microbiota at population and community scales.

This article is part of the theme issue ‘Towards a toolkit for global insect biodiversity monitoring’.

## 1. Introduction

Insects are the Earth's most diverse group of eukaryotic organisms [1]. They live on all continents, inhabit most environments, and fulfil a diverse array of crucial roles for ecosystems and humans. However, the recent magnitude and pace of ongoing global insect declines has made it clear that we cannot take these services for granted [2]. The accurate characterization of the shifting patterns of insect diversity, distribution and functions has become one of the priorities of biological research in the era of anthropogenic changes. However, to fully understand insect abundance, distribution and dynamics, we must comprehensively describe relevant drivers of insect populations and communities. Among those least understood are insects’ relationships with their symbiotic microorganisms.

The tremendous diversity of insects is reflected in at least comparable diversity of microbial symbionts. Different functional categories of symbionts have diverse and often striking effects on the life-history traits of their insect hosts, influencing their biology in different ways. Through these effects, they have played pivotal roles in insect evolutionary success and diversification, especially in exploring new food niches and responses to environmental challenges [3,4]. Through their effects on the biology and populations of keystone species and the ability to transmit and express their effects across species, they are likely to affect entire communities [5,6]. In the face of ongoing, rapid environmental changes and global loss of insect biodiversity [2], understanding the role of microorganisms in insects' biology and adaptation is becoming particularly urgent.

**Figure 1.**
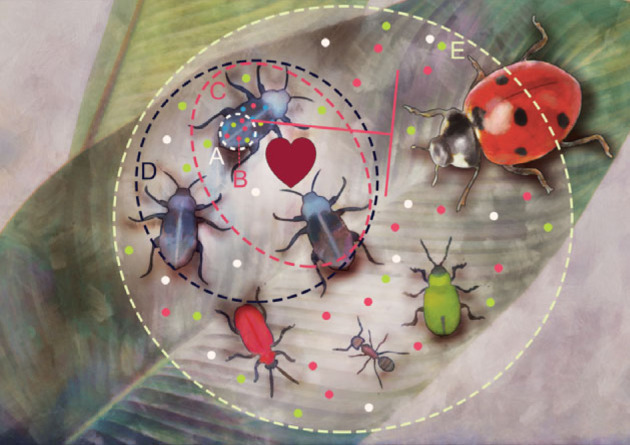
Insect–microbe symbioses are studied from different angles. A, The microbiome perspective concerns microbial communities’ composition and function across individuals, populations or species. B, The symbiont's perspective concerns specific microbial clades, focusing on their distribution, transmission, genomic evolution or functions. A distinct level of investigation concerns cellular and molecular mechanisms of host–symbiont interaction. C, The host perspective addresses questions about how symbionts affect insect life-history traits and functions. D, The population perspective concerns symbiont effects on insect populations—performance, functions, genetic diversity and evolutionary potential. E, The community perspective addresses questions about symbiotic host's interactions with other species in the community, and thus symbionts' indirect effects on community processes, composition and functions. Levels D and E are the focus of this review.

The broad implementation and rapid development of DNA-based techniques has sped up the characterization of insect–microbe associations, from various angles (figure 1). More often than not, the primary focus of the investigation has been on the microbes: microbial community composition or microbial clades’ distribution across host species or populations. Experiments have targeted microbial effects on host life-history traits and molecular mechanisms of symbiosis. Yet, we have a limited understanding of symbiosis-related processes above the individual level. Microbes other than pathogens are rarely considered a force capable of influencing insect populations and communities, and we lack answers to critically important questions at these scales:

— How are the natural populations of insects – their abundance, distributions, genetic diversity and patterns of interactions with other species – affected by microbial symbionts?
— How do symbiotic microorganisms influence populations of species other than their hosts – how common are such effects, and what are their mechanisms, directions and magnitude?
— To what extent do microbiota affect the composition and functioning of natural insect communities?
— Do microbes routinely serve as a means of transmission of ecologically relevant traits, such as the ability to feed on alternative food sources, protection against natural enemies and abiotic stressors, across insect species?
— Is microbial symbiosis a common mechanism of response and adaptation to environmental challenges and opportunities, including those of the Anthropocene era?
— Do microbes significantly influence the processes and patterns of insect biodiversity declines?
— How can we use information about microbial symbioses to aid agricultural, biomedical, conservation and other efforts?
— How can we cost-effectively characterize and monitor symbiont communities and their effects at population and community scales?

This review aims to summarize our knowledge of the many ways and levels at which microbial symbionts could influence insects. We will focus on how these effects can manifest within populations and communities, and outline approaches for studying microbiota at such scales (see box 1).

Box 1. **Glossary**

— *Symbiosis* - a close long-term relationship among different species, regardless of the nature of interaction among the partners: mutualistic, commensalistic or parasitic [7]. In insect–microbe symbioses, the insect partner is referred to as the *host*, and microbes as *symbionts*.
— While the terms *microbiota* and *microbiome* have often been used interchangeably, the term ‘microbiota’ should be reserved for describing the assemblage of microorganisms—Bacteria, Archaea, Protozoa, fungi, algae—inhabiting a well-defined habitat. The term ‘microbiome’ extends to the theatre of activity for these assemblages: microbial structures, metabolites, mobile genetic elements (e.g. transposons, phages and viruses) and relic DNA [8].
— *Horizontal and vertical transmission* are two primary means of symbiont transmission across host individuals. Vertical or maternal symbiont transmission occurs from the mother to her offspring. Horizontal transmission occurs between unrelated individuals from the same or different species.
— *Endosymbionts* are symbiotic microorganisms living within the host body cavity or hemocoel (within hemolymph, tissues, or inside cells). Symbiotic microorganisms living outside the body cavity (within the gut lumen, on the cuticle, or in various glands) are sometimes referred to as *ectosymbionts*, but this term can be confusing when referring to internally localized gut microbes. The terms *intracellular*/*extracellular* largely overlap with the endo/ecto classification, and may be more intuitive.

## 2. The functional diversity of insect symbioses

### (a) Microbial symbionts are functionally diverse

To understand the effects of microbes on insect biology, it is essential to be aware of the diversity of host–symbiont interactions. This diversity includes functional variation among symbionts and differences among insects in their reliance on symbionts. The traditional classification of insect symbionts was based on microbial localization in host tissues, specifically highlighting endosymbionts—residing within the body cavity—in hemolymph or intracellularly, within dedicated tissues [9], and forming either obligate nutritional or facultative associations. They are contrasted with microbes residing outside of the host body cavity, within the gut lumen or on the external cuticle. Recently, Perreau & Moran [10] proposed to classify symbioses as ‘Open’, ‘Closed’ or ‘Mixed’, based on transmission mechanisms and the stability of host–symbiont associations. Hammer *et al*. [11] emphasized both the stability (specialized versus transient) and the nature of the relationship (beneficial, neutral or harmful). When discussing symbioses that affect insect biology and evolution, we will primarily refer to three categories, combining aspects of those described above:

— *‘Closed’ nutritional symbioses* comprise obligatory endosymbionts like those in the sap-feeding hemipteran clade Auchenorrhyncha [12], and strictly maternally transmitted gut symbionts in insects such as *Cassida* leaf beetles [13]. These strictly heritable microbes are now essential to hosts specialized on nutrient-limited foods. Generally regarded as stable over a long time, these associations may change through symbiont replacement or complementation, potentially shifting host biology [14,15].
— *‘Mixed’ facultative endosymbionts* comprise multiple clades of bacteria that inhabit insect haemolymph and tissues and can influence many of their life history traits, particularly those related to defence and reproduction [16–20]. Through such effects, combined with the ability to transmit both vertically and horizontally, they can spread rapidly within populations and move within communities.
— *Host-adapted extracellular symbionts* inhabit the gut lumen, cuticle or proximate environment of the insect, often forming multi-species communities [21,22]. They may be transmitted from parents to offspring, socially, or acquired from the environment each generation, and can have defensive and nutritional roles.

Delimiting these and other categories, including pathogens or transient microbes, or assigning a particular microbe to one, can be challenging. Adding to the confusion are the facts that there are intermediate states, that individual microbes may be evolutionarily transiting among categories, and that even closely related microbes sometimes fall into different categories [23]. For example, insect nutritional endosymbionts often seem to be derived from bacterial or fungal opportunists or pathogens in the genera *Sodalis*, *Ophiocordyceps* and others [24–26].

### (b) Insects differ in their reliance on symbionts

**Figure 2.**
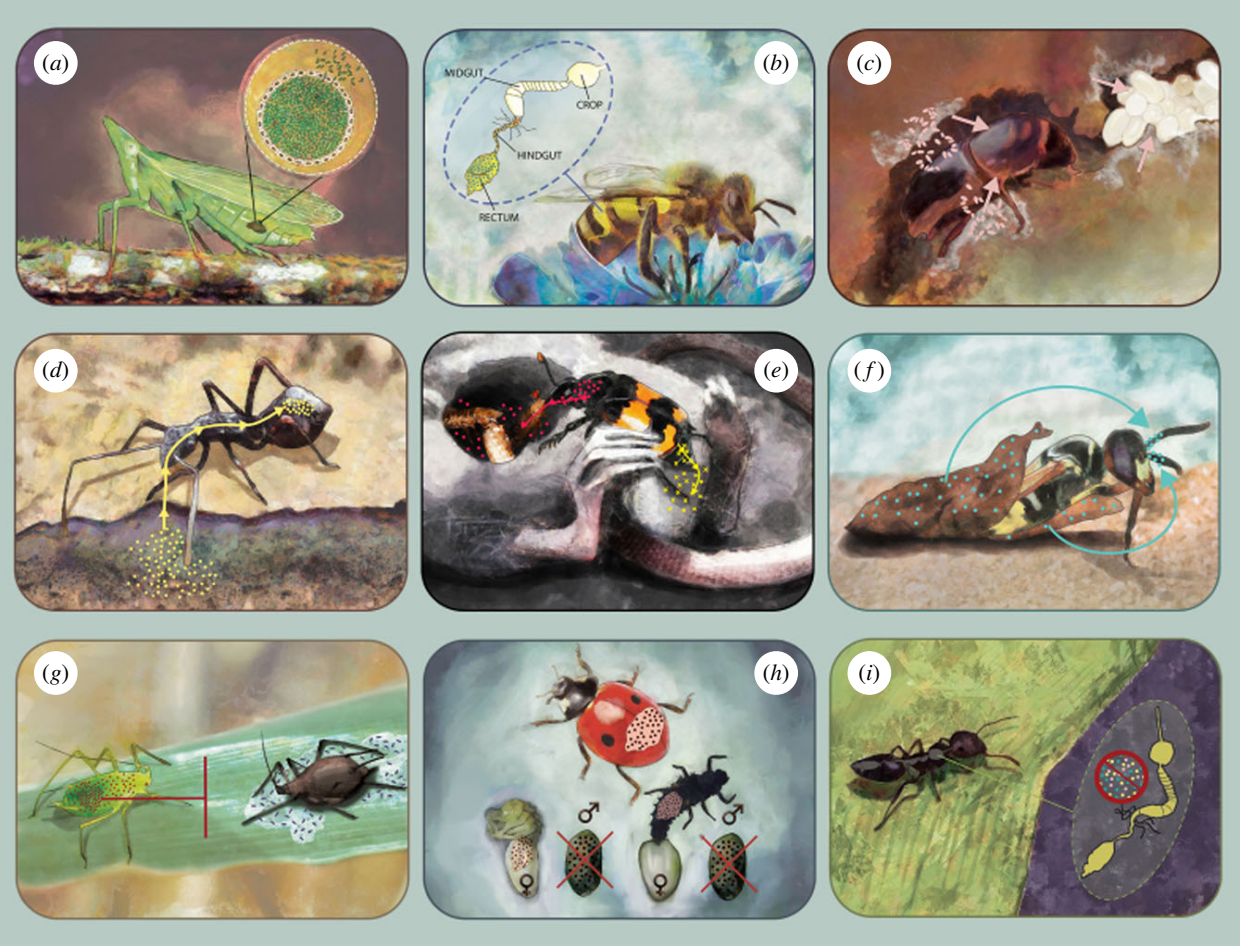
Examples of insects that rely on symbionts to different extent and for different functions. (*a*) Planthoppers are among insects feeding on imbalanced diets that obligately depend on nutritional supplementation by specialized microbes that live within dedicated insect tissues and are transmitted transovarially across generations. (*b*) Many insects rely on complex, structured, reliably transmitted gut microbial communities that provide nutritional and defensive functions. In honeybees, such gut microbiota date back at least 100 Myr. (*c*) Ambrosia beetles are among the species that culture specialized fungi. Inoculated within tunnels that beetles construct in living trees, fungi are not only critical to overwhelming tree defenses but also serve as the beetles’ only food source. (*d*) Some insects depend on microbes acquired from the environment each generation. In *Riptortus* bean bugs, *Caballeronia* symbionts, acquired by nymphs from the soil, provide nutrients and can confer other benefits, including pesticide detoxification. (*e*) Controlling the microbial community in the environment can be essential for nutrition and safety. Burying beetle adults inoculate carcasses that their larvae develop in with their microbiota and provide them to larvae while spreading antimicrobial compounds that help control harmful bacteria. (*f*) Insects' reliance on protective symbionts can lead to specific adaptations and long-term co-diversification of partners. In beewolves, *Streptomyces* that protect cocoons from fungal pathogens are transmitted within dedicated antennal glands. (*g*). Symbionts often protect insects against a variety of environmental challenges. For example, several facultative endosymbionts protect pea aphids against a specialized fungal entomopathogen. (*h*) Insects commonly have their reproduction affected by symbionts—benefitting microbes, but not necessarily the hosts. For example, in a ladybird *Adalia bipunctata*, transovarially transmitting facultative endosymbionts kill male embryos, resulting in more resources for females. (*i*) Many insects do not seem to rely on specialized microbes. Ants in the genus *Crematogaster* are among those lacking observable amounts of microbiota within their digestive tract.

Insect clades and species differ in how reliant they are, and how affected they are, by microbial symbionts (figure 2). Strict dependence is most often linked to nutrition, with insects adapted to nutrient-imbalanced foods such as plant sap or blood frequently relying on specialized, heritable microbes residing in the gut or within dedicated organs and producing essential amino acids, vitamins or enzymes [27–29] (figure 2*a*). Likewise, we expect strict reliance in insect clades that harbour conserved multi-species gut microbiota that co-diversify with hosts. Pollen-feeding corbiculate bees, wood-feeding termites, herbivorous *Cephalotes* ants and seed-feeding heteropterans all associate with such conserved gut microbiota, socially or maternally transmitted for tens of millions of years and shown to contribute to their hosts' nutritional needs [30–33] (figure 2*b*). Also, many herbivores seem to rely on certain microbes for the degradation of specific toxins present in the diet [15,34]. A distinct type of mutually obligate nutritional association is between insects such as leafcutter ants, fungus-growing termites and diverse wood-boring beetles, which all associate with specialized fungi that they farm as the sole or primary source of nutrition [35–38] (figure 2*c*).

Related categories are insects which depend on microbes for nutrition, but which are more versatile in their partner choice. Heteropterans (superfamily: Pentatomoidea) depend on nutrient provisioning for maternally transmitted gut bacteria, but different populations associate with distantly related bacteria [39]. A few mutations can turn even *Escherichia coli* into a suitable symbiont [40]. For nutrients, coreid and lygaeid bugs rely on *Caballeronia* (previously *Burkholderia*) symbionts, which each generation acquires from soil [41,42] (figure 2*d*). The reproductive success of burying beetles, whose larvae develop in vertebrate carcasses, also depends on bacterial communities with somewhat variable composition [43,44] (figure 2*e*).

Some insects obligatorily depend on specific, co-adapted microbes for defence. Beewolves (Hymenoptera, genus *Philanthus*) are protected from soil-inhabiting fungal pathogens during the pupal stage by specialized, maternally transmitted Actinobacteria within cocoons [45,46] (figure 2*f*). Leafcutter ants also rely on Actinobacteria (genus *Pseudonocardia*) for controlling fungal parasites threatening their symbiotic fungi [47]. In the psyllid genus *Diaphorina*, its symbiont *Candidatus* Profftella bears many characteristics of an obligate endosymbiont, including the ability to produce vitamins. Nonetheless, a large portion of its reduced genome comprises genes for synthesizing diaphorin, a polyketide toxin providing wide-spectrum protection against natural enemies [48]. There are also known cases of insects' dependence on a symbiont for reproduction, as shown in the hymenopteran genus *Asobara* [49].

However, many insects do not depend on microbes, and facultative infections are maintained through a balance of diverse benefits and costs—as discussed in the next section. Benefits are often linked to nutrition or protection against various natural enemies and abiotic stressors (figure 2*g*). Costs may be linked to the symbionts’ maintenance or over-replication, but also to reproductive manipulation (figure 2*h*). Finally, some insects may only occasionally associate with microbes—lacking host-specialized microbes altogether [11]. For example, ant genera differ by orders of magnitude in gut bacterial densities, and in many of them, bacterial amounts are near detection thresholds of standard techniques [50] (figure 2*i*). In these cases, low gut bacterial abundances may be due to a reduced need for nutritional supplementation thanks to balanced diets, limited opportunities for microbial colonization and/or effective microbial control mechanisms. Many other insects may be in a similar position [11], but estimates of numbers are lacking. Also, insects with no nutritional endosymbionts nor specialized gut bacteria may still be prone to at least occasional colonization by facultative endosymbionts, pathogens or transient microbes.

## 3. Non-essential symbionts' effects on insect life history traits

Symbioses essential to hosts may not necessarily be fixed, and symbiont replacement could provide hosts with novel capabilities and functions [4,51]. For example, a swap of a specialized gut symbiont between related species enabled the kudzu bug (*Megacopta cribraria*) to feed on soybeans, rendering it an agricultural pest in its introduced range [52]. However, symbionts that form facultative, non-essential associations can alter insects’ life-history traits and biology—in particular, nutrition, defence and reproduction—in a more dynamic manner. Controlled laboratory and field experiments have pinpointed these effects (table 1).

**Table 1.** Examples of non-essential microbial symbionts’ effects on insect life-history traits.

effect	examples
a shift in performance on alternative diets and extension of the dietary range	*Arsenophonus* in the cowpea aphid [53]
	*Enterococcus* in fall armyworm caterpillars [54]
	the microbiome of the Colorado potato beetle [55]
supplementation on suboptimal diets	*Bombella apis* in honeybee larvae [56]
	*Lactobacillus plantarum* in *Drosophila* larvae [57]
protection against predators	*Pseudomonas* in rove beetle *Paederus* sp*.* [58]
	facultative symbionts of pea aphids [59,60]
protection against fungal pathogens	*Regiella* and other facultative endosymbionts in the pea aphid [61]
	*Burkholderia* in the cuticle of *Lagria* beetles [58]
	facultative endosymbiont *Rickettsia* in whiteflies [62]
	*Streptomyces* in beewolf cocoons [45]
protection against parasitoids	*Hamiltonella* in different aphid species [63]
	*Spiroplasma* in *Drosophila hydei* [64]
protection against entomopathogenic nematodes	*Spiroplasma* in *Drosophila neotestacea* [65,66]
	fungal symbiont in *Sirex* wood wasps [67]
	scarabid beetle (*Melolontha melolontha*) microbiome targeting mutualistic symbionts of nematodes [68]
protection against microeukaryotic parasites	bumblebee gut microbiota against trypanosomatid *Crithidia bombi* [69]
protection against bacterial pathogens	larval microbiota in Japanese honeybees protect against bacterium *Paenibacillus* larvae [70]
	*Morganella* and *Providencia* protect carrion beetles against *Serratia*-induced mortality [43]
protection against viruses	*Wolbachia* in *Drosophila* [71], mosquitoes [72] and brown planthopper *Nilaparvata lugens* [73]
protection against heat shock	*Regiella, Fukatsuia* [74] and *Serratia* [75] in the pea aphid
	*Wolbachia* in *D. melanogaster* [76]
protection against chemical stressors	soil-acquired *Caballeronia* in the bean bug and related heteropterans [77]
	egg microbiome in chironomids [78]
	coffee berry borer microbiome degrades caffeine [79]
overall increase in reproductive fitness	facultative endosymbiont *Rickettsia* in whiteflies [80]
	*Wolbachia* wRi in *Drosophila simulans* [81,82]
reproductive manipulation	diverse facultative endosymbionts including *Wolbachia*, *Rickettsia*, *Spiroplasma*, *Arsenophonus* and *Cardinium*, induce feminization, male killing, parthenogenesis or cytoplasmic incompatibility in diverse insects [83,84]
induction of dispersal	*Regiella* reduces winged morph induction under crowded conditions in pea aphids [85]

### (a) The diversity of facultative symbionts' effects

Non-essential microbial symbionts can also alter insect nutritional biology, by enabling expansion or shift of the dietary range, or by reinforcing specialization, potentially leading to reproductive isolation and speciation [53]. For example, in pea aphids and cowpea aphids, facultative endosymbionts have been shown experimentally to improve the fecundity on some of the possible host plants [86,87].

Symbionts may also contribute to nutrition in more specific ways, for instance, by buffering the effects of malnutrition in *Drosophila* fruit fly and honey bee larvae [56,57]. Likewise, in *Drosophila melanogaster*, *Wolbachia* increases the fecundity on diets with suboptimally low or high iron concentrations [88].

Among the most striking effects of insect symbionts is protection against a wide variety of natural enemies. In multiple insect systems, different symbiont clades have been shown experimentally to protect against predators, parasitoids, entomopathogenic nematodes, fungal entomopathogens, parasitic microeukaryotes, bacterial pathogens and viruses [16–18,20] (table 1). These effects can be manifested at different stages of insect–natural enemy interaction: from affecting the detection and attack or infection success through promoting biomechanical, chemical and immunological defences to disrupting enemy-induced phenotypes that facilitate infecting further hosts [61,89,90]. Experiments have consistently shown an increase in survival and/or reproduction of individuals carrying protective symbionts and a decrease in the success and fitness of natural enemies [91–93].

Likewise, symbionts can also confer protection against a wide range of environmentally relevant abiotic stressors. Facultative endosymbionts have been shown to protect their insect hosts against heat [74,75,94,95], water loss and desiccation [92], pesticides [77], and heavy metals [96] (table 1). On the other hand, symbionts’ susceptibility to these factors can be the insects' weakness, as demonstrated by heat-disrupted beneficial symbioses of aphids and stinkbugs [97–99], or herbicide-disrupted symbioses of bees [100].

Another important category of symbionts’ effects is the manipulation of insect reproduction [83]. There are four distinct strategies of reproductive manipulation, each induced by strains of several different bacterial genera, and each reported from a wide range of hosts [19]. The feminization of genetic males leads to their development into functional females that can pass on the infection to their offspring. Male killing causes the death of male embryos, increasing the share of the local resource pool available to females and translating into their improved survival, growth and, ultimately, reproductive output. Another strategy is parthenogenesis induction, where unfertilized eggs develop into females. Finally, cytoplasmic incompatibility leads to high mortality of embryos sired by symbiont-infected males unless the same symbiont strain also infects the mother, reducing non-infected females' reproductive success. While benefitting the symbiont and facilitating its spread in a population, all these effects generally occur at the expense of at least some components of the insect fitness: the reproduction through females is generally improved, but the reproduction through males suffers [83]. Symbionts can also have more nuanced reproductive effects, such as altering the induction of sexual reproduction in cyclically parthenogenetic aphids [101].

The symbiont also affects insect host life-history traits directly related to reproductive fitness, including juvenile survival, time to reproduction, lifespan and fecundity. These effects can range from highly detrimental to strongly positive [102], depending on environmental conditions, genotypes, and likely other variables. Some other symbionts’ effects are harder to categorize. For example, at low population densities, aphids tend to develop into wingless morphotypes with shorter development time and higher fecundity but limited mobility, while under crowded conditions, they are more likely to develop into less-fecund winged forms [103]. Facultative endosymbionts can decrease aphid sensitivity to crowding in regard to winged morphotype induction, thus decreasing their propensity to migrate [85].

It is important to note that microbial symbiont strains often simultaneously alter different life-history traits of their hosts. An example is the combination of reproductive manipulation and antiviral effects conferred by different strains of *Wolbachia*, which has resulted in their widespread adoption for controlling mosquito-vectored dengue or related viruses. Regardless of the primary effect, symbiont infections are expected to alter host traits such as development time, fecundity and longevity, as shown repeatedly for different host–microbe associations [81,102,104]. These effects are important for determining the net adaptive value of the infection. Overall, under specific conditions, the net effects of carrying a particular symbiont by a given host genotype may reflect a fine balance of different costs and benefits, and the costs of infection may often exceed the benefits [105,106].

### (b) The effects of symbionts depend on the context

Symbionts’ phenotypic effects can be expressed in different host genotypes and species. For example, facultative symbionts and their effects can be artificially transmitted across aphid and *Drosophila* clonal genotypes and species [107,108]. Likewise, the use of *Wolbachia* wMel for dengue control is possible thanks to its similar reproductive and antiviral effects in original (*Drosophila*) and new (*Aedes*) hosts [109]. On the other hand, the symbiont effects can vary among genotypes and species of symbionts, their insect hosts and natural enemies that symbionts protect from [63,110,111]. The diversity of fungus- or parasitoid-protective facultative endosymbionts in pea aphid populations was explained by the specificity of their protection against diverse natural enemy genotypes present in the environment [61,105]. Specificity has also been demonstrated in reproductive manipulation, revealing cases where host genotypes vary in susceptibility to manipulative symbionts [112], and where the same symbiont strain manipulates alternative hosts in different ways [113]. The net effects of carrying a symbiont may thus depend on environmental conditions—the selective pressure imposed by factors that the symbiont protects against. The value of carrying a symbiont that protects against heat shock or a particular natural enemy is obviously greater in populations exposed to heat or these natural enemies.

Symbiont effects on hosts can also be affected by environmental conditions. For example, elevated temperatures reduce parasitoid protection conferred to aphids by *Hamiltonella* [114], or antiviral protection provided by *Wolbachia* [115]. Environmental conditions can also affect host–symbiont interactions indirectly [95]: elevated temperatures can reduce *Wolbachia* maternal transmission efficiency [116], leading to the loss of any protective or reproductive effects it may have. Last but not least, symbionts can alter each other's effects in co-infections. In aphids, *Fukatsuia* extends the thermal range of *Hamiltonella*-conferred parasitoid protection [74], *Hamiltonella* may ameliorate negative fecundity effects of *Rickettsia* without decreasing its fungal protection strength [107], and *Regiella* and *Hamiltonella* influence each other's defensive and fecundity effects in complex ways that vary among host genotypes [117]. Symbionts can also affect each other's transmission efficiency [118] (see box 2).

Box 2. ***Wolbachia* as a widespread symbiont clade with diverse infection effects.**

The alphaproteobacterial genus *Wolbachia* is the most broadly distributed insect symbiont, with at least 17 recognized clades (supergroups) colonizing diverse insects, other arthropods and filarial nematodes [119]. *Wolbachia* infects about half of all insect species, but the infection prevalence varies among and within species, with heterogeneity observed across populations and over time [113,120,121]. Its associations with insects range from facultative to obligate, covering the spectrum from parasitism to mutualism [80].

*Wolbachia* has historically been regarded as a reproductive parasite, manipulating hosts' reproduction in four distinct ways: cytoplasmic incompatibility, male killing, feminization of genetic males, or parthenogenesis induction [83,121]. In some cases, exemplified by a hymenopteran parasitoid *Asobara tabida*, its tight integration into host oogenesis has made it necessary for reproduction—a sort of indispensable parasite [122]. At the same time, *Wolbachia* can have important beneficial effects. Its protective effects, especially against viruses [71,123], are the foundation of extensive experimental work on *Wolbachia*-driven control of mosquito-vectored dengue and related viruses [109], or planthopper-vectored plant viruses [73]. Recently, *Wolbachia* was also shown to protect aphids against a fungal pathogen [124]. In bed bugs, it is an essential nutritional mutualist providing B vitamins required by these obligatory blood feeders [125]. More subtle and non-essential nutritional benefits have been reported from *Drosophila melanogaster*, through the symbiont's apparent effects on iron metabolism [88]. Finally, *Wolbachia* has been linked to host thermal preferences and performance, as suggested by geographic gradients in its prevalence [116].

Like other facultative endosymbionts, *Wolbachia* transmits mainly maternally (vertically) with high fidelity but is also capable of horizontal transmission within and across species [113]. The presence of nearly identical strains in different host species [126] and the lack of congruence between host and symbiont phylogenies [127] highlight host switching as an important aspect of *Wolbachia* biology, leading to the transmission of its phenotypic effects across host species [113]. The combination of reproductive manipulation, fitness benefits, and alternative transmission means thus provides avenues for *Wolbachia*'s rapid spread within and among species [126,128], affecting population- and community-scale processes and patterns.

## 4. How symbionts affect insect populations and species

### (a) Symbiont effects drive spatial and temporal variation in infection prevalence

Generally, once a heritable symbiont with a significant positive net effect on fitness is established in a population, we would expect the infection to spread along with its ecologically relevant effects on hosts [129]. These processes may lead to structuring beneficial symbiont infections alongside environmental gradients relevant to symbiont effects, such as natural enemy pressure, temperature or available food [62,95,116,130]. Further, in species with short generation times, seasonal changes in environmental conditions should alter the balance of infection costs and benefits, likely promoting different types of infections at different times of the year and driving seasonal fluctuations in the prevalence of different symbionts within populations [131–134]. These processes could lead to local or seasonal spread of adaptive phenotypes, increasing the overall population performance under specific conditions. Because of their effects, symbionts—especially facultative endosymbionts—have been described as a pool of horizontally transmitted vectors of ecologically significant traits that enable rapid response and adaptation of species to environmental challenges and opportunities [5,6]. The symbiont's primary fitness benefits, plus any additional effects on other life-history traits, could rapidly shift the host species' ecological niche [51].

The link between symbionts’ effects on individual life-history traits and their effects on host lines' competitive performance and population structure has been shown experimentally using semi-controlled population cages with infected and uninfected lines of species such as *Acyrthosiphon pisum, Bemisia tabaci, D. melanogaster and D. neotestacea.* In the presence of natural enemies, symbionts’ protective effects have led to the spread of the infection [62,135–137]. In two aphid species, symbiont-conferred protection was demonstrated in the field, but apparently counterbalanced by infection trade-offs [105,106].

**Figure 3.**
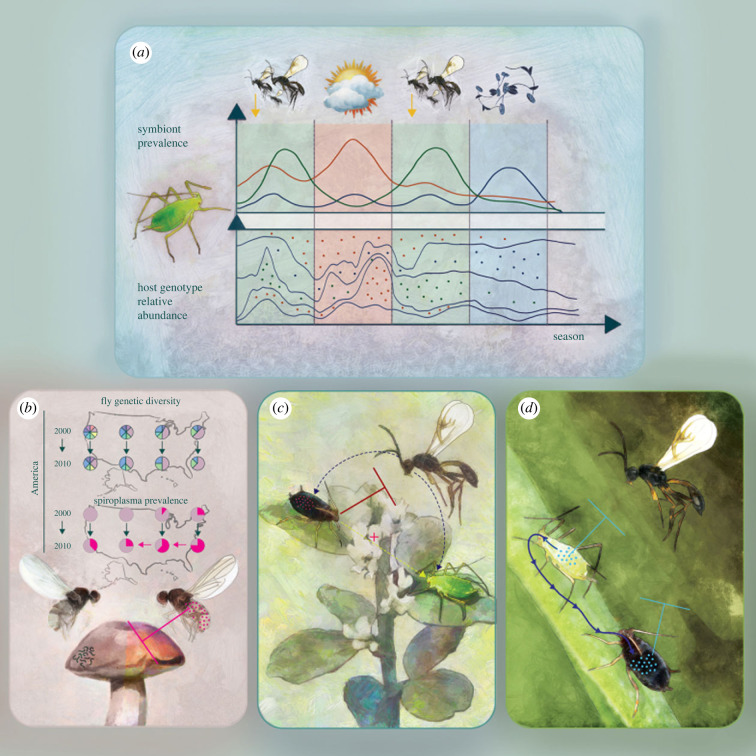
Examples of symbiont effects on insect populations and communities. (*a*) Environmental pressures that aphid facultative endosymbionts can protect against, including the pressure of parasitoids, pathogens and heat, vary throughout a season, promoting certain symbiont associations. The shifting prevalence of alternative symbionts affects the competitive balance among clonal lineages that carry them, affecting population structure. (*b*) Rapid, continent-wide spread of nematode-defensive *Spiroplasma* symbiont in mushroom-feeding *Drosophila neotestacea* has resulted in the loss of host genetic diversity across populations. (*c*) Defensive symbionts can protect their hosts directly but also have a range of indirect effects on the same or other species. For example, they can negatively affect populations of parasitoids that also attack other species, thus indirectly protecting these species. (*d*) Facultative endosymbionts can transmit horizontally among species in a community and express the same effects in novel hosts. Hence, transmitting a parasitoid-protective symbiont to a new host species can make that species also resistant to parasitoids.

Patterns consistent with directional selection for maintaining certain symbionts were also reported from natural insect populations sampled across environmental gradients. For example, aphids from more northerly regions of Japan, characterized by higher humidity and thus more favourable conditions for fungal pathogens, carried pathogen-protective facultative endosymbiont *Regiella* more often [131]. On the other hand, comprehensive symbiont-monitoring efforts in the eastern US indicated that aphid facultative endosymbioses can be very dynamic. The prevalence of facultative symbionts in pea aphid populations changed throughout the season, sometimes, but not always, correlating with environmental pressures the symbionts protect against [131,132,134] (figure 3*a*). This suggested that the symbiont prevalence is determined by a complex balance of infection costs and benefits, fluctuating over time in response to natural enemy pressures, temperatures and other variables.

Strong symbiont-induced directional selection has also been reported from other systems. Over the last two decades, the facultative endosymbiont *Spiroplasma* which protects fungivorous *Drosophila neotestacea* against an important natural enemy, the entomopathogenic nematode *Howardula aoronymphium*, has spread across the northern US (figure 3*b*) [65,66]. The symbiont spread was linked to substantial changes in the host's population structure, including shifts in the relative abundance of mitochondrial variants across the surveyed range. Similarly, anti-fungal properties seem to have driven a rapid spread of *Rickettsia* across Chinese populations of the sweet potato whitefly *B. tabaci*, following the spread of the entomopathogenic fungus the symbiont protects against [66]. In turn, the spread of *Rickettsia* in invasive whiteflies from the southwestern US [138] seems to have been driven by a combination of reproductive manipulation and positive effects on female fecundity and development time [80]. Reproductive manipulation, at least sometimes combined with fitness benefits, has promoted the spread of symbionts such as *Wolbachia* in many other species [65,113].

Non-heritable symbionts can also alter host biology and performance in response to human-induced pressures. *Caballeronia* strains acquired from the soil by juvenile stinkbugs from pesticide-treated areas frequently have the ability to detoxify these pesticides, making their hosts resistant and thus leading to symbiont-induced resistance within populations [77]. Symbiont-induced pesticide resistance was also reported from other insect systems [139].

### (b) Heritable symbionts shape insect populations and evolution

In the case of symbionts that primarily transmit maternally, symbiont spread within a population occurs through improved performance of host lines that carry these symbionts. This changes the population's genetic makeup, leading to a symbiont-driven decrease in the host genetic diversity in a population [66,140,141]. Such effects may extend into selective sweeps, where symbiont-carrying host lines initially representing a small proportion of genetic diversity end up dominating the populations. Loss of genetic variation may negatively affect insects' ability to respond to the changing environment through recombination and natural selection acting upon their own genomes [29,142]. Barriers to within-population gene flow caused by reproductive manipulation may have similar detrimental effects on the hosts’ adaptation potential through their nuclear genome evolution. Further, the reproductive manipulation-driven decrease in the host's effective population size would increase the impact of stochastic processes.

In the longer term, symbiont-induced barriers to gene flow could facilitate and drive speciation. Among the best-studied examples is the Central and South American *Drosophila paulistorum* species complex, where isolation among reproductively isolated but often sympatric ‘semi-species’ is caused by *Wolbachia* [143]. Pre- and post-mating isolation among them seems to be driven by the symbionts' effects on host gene expression, altering pheromone production and reception [143,144]. On the other hand, symbionts’ can also influence insect evolution through horizontal gene flow, whether serving as the original source of bacterial genes integrated into the host genome or by mediating gene transmission among different organisms [145].

Although heritable symbionts can open up new avenues for insects' speciation and diversification, they can also limit the host's range and ecological niche due to symbionts’ constraints. Heat sensitivity of some nutritional symbionts is thought to limit the distributions of clades such as aphids [98,99]. The reliance on an obligate symbiont that undergoes genomic degeneration could lead to the extinction of evolutionary lineages [29,97].

## 5. Symbiont effects within multi-species communities

### (a) Symbionts influence their host's interaction with other taxa

Symbiont effects may extend to other organisms with which the focal species interacts: predators, parasites, parasitoids, competitors. Among the most intuitive indirect symbiont effects are those that protective symbionts have on their hosts' natural enemies. Symbiont-conferred protection should decrease natural enemies’ performance, negatively affecting their population processes. Indeed, in simple experimental communities, introducing defensive symbionts has led to the decline and, ultimately, extinction of entomopathogenic nematodes attacking *D. neotestacea* flies [136], or parasitoids attacking aphids [146]. We could expect the same types of effects against other categories of natural enemies.

However, the outcomes can be complicated by the specificity of protection. In aphids, strains of their defensive endosymbiont *Hamiltonella* vary substantially in the degree of protection they confer against parasitoid genotypes and species [146,147]. A high prevalence of a symbiont conferring an effective protection against a particular parasitoid species should lead to the decline of that parasitoid unless compensated by its rapid behavioural or genetic responses. Conversely, the shifting abundance or pressure by different parasitoids may change the adaptive value of hosting alternative defensive symbionts and, therefore, their prevalence in the host population. An increase in pressure by a certain parasitoid genotype would promote the spread of symbiont genotypes that protect against that particular natural enemy, which would then lead to its decline [129,131].

Analogously, symbionts could affect other types of interactions among host insects and other organisms. If a symbiont influences its herbivore host's feeding on a certain food plant—whether directly or indirectly, by altering the plant's anti-herbivore defences [148]—changes in infection patterns within the herbivore population could affect the plants' performance and in the longer term their abundance. This would alter the adaptive value of hosting the symbiont for the herbivore, influencing infection prevalence, the herbivore's dietary range and the pressure on the food plant.

### (b) Cascading effects of symbionts

The effects of symbionts can be cascading, influencing further species across the trophic network [5,6]. Species in a community are typically attacked by multiple natural enemy species—and correspondingly, predator or parasitoid host ranges frequently encompass multiple insect species [149]. A defensive symbiont in insect species A suppressing its natural enemy may then indirectly protect species other than A that are attacked by the same natural enemy [136] (figure 3*c*). On the other hand, we could also envision a behavioural shift in preference of the natural enemy towards species other than A, and an increased pressure on them. Either way, any substantial symbiont-mediated indirect effects on populations of other species will affect an even broader range of species that interact with them. That way, the effects of a defensive symbiont in a single species could reverberate through the whole community. These processes have been demonstrated in simple experimental communities comprising different aphid and parasitoid species. Introducing a defensive symbiont into one of the aphid species has substantially affected populations of other species, sometimes leading to their extinction and the subsequent cascading community collapse [150]. We would expect such effects to be more subtle in spatially complex and heterogeneous natural ecosystems. Nevertheless, if the biology of keystone or outbreaking species is significantly affected by symbioses, one would expect substantial effects at the community level.

Food plants of herbivorous insects can also mediate symbionts' indirect effects on communities. The alteration of plants’ anti-herbivore defences by herbivores' symbionts could affect the performance of both the original herbivore and other herbivorous species [151]. Changes in the symbioses of one herbivorous species may well thus affect other species feeding on the same plant. Further, by producing volatile compounds, plants can communicate with each other, leading to the induction of anti-herbivore defences in plants other than the one originally attacked. Likewise, through volatile production, plants can attract herbivores’ natural enemies [89]. By attenuating the production of these volatiles, herbivores' symbionts can thus indirectly protect not only their own hosts, but also alter the entire herbivore and natural enemy community in the vicinity [89].

### (c) Symbionts as vectors of ecologically important traits across species

A completely different take on symbiont roles in natural ecosystems is by considering their horizontal transmission within multi-species communities. As discussed, extensive phylogenetic evidence exists for facultative endosymbionts’ transmission both within and across insect species, and their diverse effects can be expressed in a range of host genetic backgrounds. This has led several authors to suggest that facultative endosymbionts should themselves be regarded as the community, at least partly independent from the communities of insects that they colonize [5,6] (figure 3*d*). Such symbiont communities could be regarded as pools of horizontally transmitted agents of ecologically relevant functions that insect species subsample in response to various environmental pressures. They have been compared to plasmids in bacterial communities—exchanged among lineages and species, inherited by daughter cells, and encoding a variety of ecologically important functions [152].

This perspective aligns well with observations for systems such as aphids and *Drosophila* flies. Aphid facultative endosymbionts can be transmitted experimentally even among distantly related species [108,153], and there are indications of symbiont transmission within and among species on ecological timescales [154,155]. The balance of infection costs and benefits in the new genetic background, under the environmental conditions experienced, determines the fates of such new infections [132]. Likewise, the discovery of nearly identical *Wolbachia* strains in *Drosophila* species separated by tens of millions of years of evolution and from different corners of the globe strongly suggests that these symbionts do indeed transmit within communities at relatively short timescales [126]. There is also evidence that microbes may transmit across more distantly related but closely interacting species, for example, co-habiting ants and their associated beetles [155].

For other insect–symbiotic systems, we have fewer indications that interspecific facultative endosymbiont transmission may occur at ecologically relevant timescales, especially across insect species that are more distantly related and functionally less similar. Recent genome-level phylogenies for over 100 *Wolbachia* strains and their diverse insect hosts do indeed reveal extensive symbiont transmission as species evolved [127]. The patterns observed in that study could have resulted from symbionts typically co-diversifying with hosts for millions or even tens of millions of years before potentially ‘jumping ship’. On the other hand, one could also imagine the same patterns resulting from symbionts transmitting among species on a yearly basis. To date, we cannot reliably distinguish among these scenarios without comprehensive and systematic sampling both within insect populations and species and across communities. Such studies will also require additional phylogenetic resolution from more marker genes.

Overall, we think that insect-associated bacteria within natural ecosystems represent a large gradient of host specificity and abilities to transmit among species. There may not be a single symbiont pool accessible for most members of the insect community. Rather, there may be multiple symbiont sub-pools transmitting more freely among related, or ecologically similar, or closely interacting species. In at least some systems, such inter-specific transmission of symbionts and the ecologically relevant traits that they encode do seem to occur on ecologically relevant timescales.

## 6. Symbionts as an unexplored means of rapid adaptation to environmental and anthropogenic challenges

The wide range of microbial symbionts' fitness effects, combined with the ability of many of them to transmit maternally and horizontally, identify symbionts as likely means of rapid response and adaptation to a range of environmental pressures. This includes natural pressures associated with environmental gradients, such as those of temperature or rainfall, or seasonally variable drivers, such as the impacts of natural enemies. It also includes pressures that have appeared or intensified due to human activities. Global climate change alters local weather patterns and increases the incidence and severity of extreme weather events such as heat waves and drought episodes [156]. The production and environmental release of toxic agricultural chemicals continues going up on a global scale despite local declines [157]. The continuing destruction, degradation and fragmentation of natural habitats is taking away living space for many organisms and disrupting species interaction networks [158,159]. Invasive species also adversely affect habitats and pressure native species through various means [160,161]. All these pressures have been listed among the most important drivers of the ongoing insect biodiversity declines [2,162]. At the same time, as discussed in the previous sections, insect symbionts can confer protection against most of these pressures in at least some insect systems.

Species can respond to novel or intensifying pressures in different ways. Responses can include behavioural changes or range shifts as well as genome evolutionary processes, including random mutations and recombination, combined with genetic drift and natural selection [163]. However, these processes are slow relative to the pace of the ongoing global changes. It is thus interesting to consider the potential for rapid adaptation through the acquisition of symbiotic microorganisms, especially facultative endosymbionts. Such infection could lead to the near-instantaneous acquisition of complex, multi-gene traits, often of direct environmental relevance [4,5,51]. The effects of the new infection may be largely independent on gene sets or traits already possessed, and the change may be heritable. Following such infection, the entire host–microbe symbiosis becomes the subject of selection—with the symbiont spreading in the population and the community alongside its host line. Such symbiont-driven adaptation could be more rapid than through evolutionary processes involving the host genome. In species subjected to very strong environmental pressures, one could imagine the spread of a protective infection to a high frequency within a few generations. The rapid continent-scale symbiont sweeps reported from *Drosophila* species [66,82] and whiteflies [62,138] exemplify the process. Following the spread, evolutionary processes acting upon the symbiont genome—much faster than those affecting the host genome—could fine-tune and improve the symbiosis further [3,81]. Also, the increased local prevalence of a protective symbiont would likely increase the odds of its horizontal transmission within species or the colonization of new host species within the community.

Confirmation that such symbiont-driven rapid adaptation indeed frequently occurs in natural ecosystems would substantially change our understanding of community function and underlying processes, especially in the face of the intensifying and speeding-up of global changes. It could also open up new avenues for species management and conservation in agricultural or natural systems. But are such processes indeed common?

To date, we do not understand the breadth, structure or nature of the symbiont pool that could plausibly be ‘sampled’ by wild insect species, that has a reasonable chance of colonizing a given host, and then form stable infections [6]. We are aware of the primary barriers to symbiont acquisition [164] but do not know their actual importance in natural systems. Through all these, we have no knowledge of how often symbionts such as *Wolbachia* may successfully colonize populations of a new host species and get a chance to spread: on the scale of days, or millennia? We do not know the most common features and effects of successful spreaders—perhaps a combination of defensive properties with reproductive manipulation? Further, for symbionts that establish a foothold in a population, we do not know what are the typical fates of these infections. How common are rapid, selective sweeps, as opposed to slow increase to intermediate prevalence or co-existence of multiple strains at low prevalence in the population? How important are the interactions with any older symbionts? What happens after a successful sweep—is it common that the fitness benefits gradually dissipate [165]?

While these questions remain open for now, we are finally in a position to resolve them. To get an accurate picture of the relevant processes and patterns, we need to embark on a much more systematic characterization of insect–microbe interactions in the wild than has been achieved so far.

## 7. How to incorporate microbiome characterization in insect biodiversity surveys?

### (a) Alternative research approaches, and what they can teach us

Studying microbiota in large numbers of wild insects requires a robust sampling plan, as well as a carefully designed conceptual, laboratory and analytical workflow which should encompass:

— Means of obtaining information about the taxonomic identity of the collected insects and, preferably, the presence and identity of parasitoids or parasites that could alter the microbial community profiles.
— Data on the presence, absolute abundance, and taxonomic identity of microbes that may colonize the insects—bacteria, but ideally also fungi, microeukaryotes and viruses.
— The ability to obtain high phylogenetic resolution for selected hosts and microbes and information about their functions.
— Labour- and cost-effectiveness of the workflow so that it is can be applied to hundreds or thousands of individuals at a time.
— The microbiome-focused workflow should be well-integrated into broader biodiversity surveys, enabling the reuse of specimens and exchange of information among levels of investigation.

So far, only DNA-based techniques provide sufficient throughput and resolution to enable microbial community surveys at the required scale. The available methods vary in the information provided, throughput and cost, and none can address all points listed above on its own. However, the comparison of their features suggests how they can be applied jointly.

*Diagnostic PCR* is a test of whether a marker region of interest, usually a portion of the 16S rRNA gene for a strain or clade of microorganisms, can be amplified from and is thus present in a sample. It is the cheapest and conceptually most straightforward method of symbiosis study, but provides a limited amount of information. Nevertheless, combined with *Sanger sequencing*, which validates their specificity and provides phylogenetic information, diagnostic PCRs have been used widely and will remain useful for addressing specific questions in the future, as demonstrated by recent high-profile studies focused on certain microbes in particular host species [62,82,132].

*High-throughput sequencing of amplicons of marker genes* such as bacterial 16S rRNA provides information on the diversity and relative abundance of sequence variants across samples. Despite known biases and challenges [166], the method is suitable for addressing broad microbial diversity and distribution questions. The simultaneous targetting of insect and bacterial markers can provide host identity and parasitoid infection information, thus serving as a robust framework for microbiota characterization [167].

*Metagenomics* is the high-throughput sequencing of the total host and microbial DNA extracted from an insect. It is the most comprehensive method, providing detailed information about symbionts' and hosts' phylogenetic relationships and functions. Unfortunately, high per-sample cost and analysis complexity have limited the implementation at scales exceeding a few dozen metagenomic datasets [21,25,168]. An interesting alternative is *target enrichment* or *hybrid capture sequencing*. In this approach, before sequencing, pre-selected phylogenetically or functionally informative regions are enriched from metagenomic libraries [169], substantially reducing the sequencing depth (and cost) needed for the sequence reconstruction. However, the method has not been widely applied in insect symbiosis research [170,171].

As these very same DNA-based approaches can be used for insect biodiversity surveys (see other contributions to this Themed Issue), we should consider how much these data could teach us about microbiota. The most popular approaches are based on high-throughput sequencing of amplicons of insect marker genes, generally mitochondrial cytochrome oxidase (I), or COI [172], whether using DNA extracted from whole multi-species samples (metabarcoding) [173], or from large numbers of individual insects (high-throughput barcoding) [172]. These COI amplicon-based approaches could serve as a helpful starting point in microbiome characterization, providing information on the identity of insects but also informing about the presence of certain microbes. Specifically, barcoding primers are known to amplify COI sequences of alphaproteobacterial symbionts *Wolbachia* and *Rickettsia* in addition to insect genes [174]. These COI data may enable symbiont detection and improve phylogenetic resolution relative to 16S rRNA, at least for *Wolbachia* [175]. Detection reliability likely varies depending on the protocols used—DNA extraction strategy, primers, sequencing platform and depth—and needs to be validated. Nevertheless, mining COI amplicon datasets generated to study insect diversity could provide a window into microbiome patterns extremely cost-effectively. The growing insect-genomic datasets can also serve as a valuable source of high-resolution information about symbioses [127,176]. On the other hand, mining symbiont information from metabarcoding or metagenomic data for mixed multi-species insect community samples can be more challenging due to difficulties in establishing host–symbiont relationships.

### (b) Combining research tools into a robust workflow

Among the challenges in interpreting modern high-throughput barcoding data is their sheer scale, sometimes encompassing tens or hundreds of thousands of specimens [177,178]. However, besides providing information on *Wolbachia* infection, such extensive collections of pre-barcoded insects could then serve as material for more systematic microbiota characterization. We have recently verified that the non-destructive HotSHOT treatment, used for obtaining DNA as a part of a cost-effective barcoding workflow [179], does not significantly alter the microbial community profile reconstruction [180]. Thus, we may use barcoding-derived species IDs to select specimens for processing using more comprehensive methods such as marker gene amplicon sequencing. That way, we can maximize the taxonomic diversity of processed samples while avoiding the oversampling of abundant species and correct for *Wolbachia* infections as a potentially important variable in determining overall microbiota composition. Similarly, marker gene amplicon sequencing data could be used to select samples for target enrichment sequencing or metagenomics.

**Figure 4.**
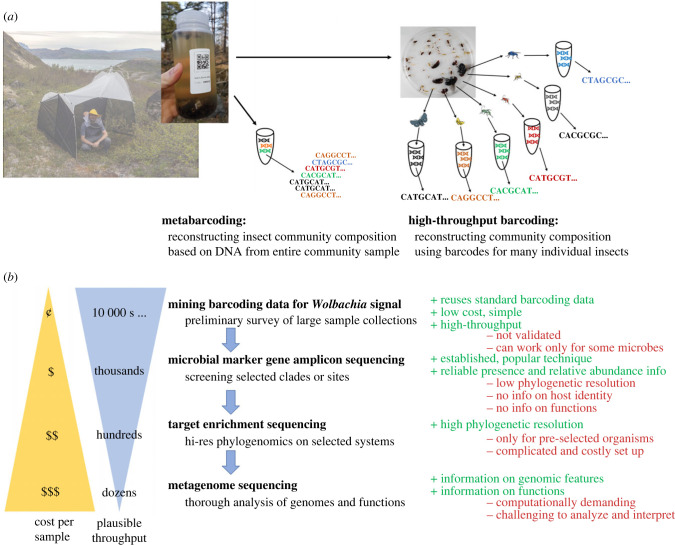
Alternative approaches to the characterization of communities of insects and their associated microbes. (*a*) The primary DNA-based approaches to insect community characterization rely on reconstructing insect marker gene sequences, either from bulk multi-species samples (metabarcoding) or for large numbers of individual insects (barcoding). (*b*) The approaches to microbiome characterization in collections of wild insects vary considerably in plausible throughput and per-sample cost. The comparison of their strengths and limitations suggests how they can be applied and combined into a workflow that combines breadth and deep insights into selected symbioses.

A comparison of alternative methods' features, including throughput, the information provided and per-sample cost, suggests how methods could be combined into an effective workflow for microbiome surveys at population or community levels and integrated into broader biodiversity surveys (figure 4). We can use insects from broad sampling efforts for high-throughput non-destructive barcoding, providing information on the taxonomic identity of large numbers of individually tracked specimens [179]. Combining barcodes and information on alphaproteobacterial symbiont infections can provide a comprehensive picture of infections with some of the most significant microbes. Simultaneously, it can guide the selection of specimens for microbial marker gene amplicon sequencing, providing a broad picture of microbial genotype and clade distribution across populations and species. These data will likely pinpoint host–symbiont associations that are of particular interest because of their distribution or putative effects on hosts. They could guide the selection of specimens for target enrichment sequencing—providing a high phylogenetic resolution that enables the detection of patterns such as the recent or ongoing horizontal transmission of symbionts across species. Finally, a subset of available metagenomic libraries could be sequenced to high coverage without the enrichment step, enabling the full reconstruction of genomic features and functions. Overall, such a multi-step workflow could start with a broad insect biodiversity characterization, reconstruction of microbiome-related processes and patterns within selected clades and species, and then focus on the biology of selected microbes.

Through rapid technological progress, more microbiome-surveying tools may soon become available. Improved reagents and protocols and laboratory automation simplify high-throughput sample processing. The orders-of-magnitude decrease in the cost of 1 Mb of sequence data since the turn of the century and the emergence and rapid improvement of long-read sequencing technologies have opened up new opportunities [181]. The speed and capacity of computational resources have improved in parallel. Arguably, we are no longer limited by technology when addressing broad questions about host-microbiome interactions using DNA-based and computational techniques. Instead, the availability of highly trained personnel and the ability to interpret the data might often be limiting factors. It is intriguing to consider whether the developments in robotics and artificial intelligence [182] might help address these gaps. These developments could help us take advantage of further improvements in the technologies listed above, along with new opportunities, including those that would not require the destructive sampling of insects in order to characterize their microbiota [183–185].

## 8. Conclusion: insect biodiversity researchers must consider microbiota

Microbial symbionts are an essential component of insect biology: we need to know their microbiota to understand insects fully. Symbionts have been critical in the evolution of insect nutrition and present-day nutritional biology, highly relevant in many species of agricultural and medical significance. Symbionts’ effects on insect reproduction are a potent means of affecting host population structure and processes such as speciation. Their diverse effects on insect life-history traits, interactions with other species, and susceptibility to abiotic factors known to strongly influence the composition of communities can shape the insects' ecological niche. The symbionts’ ability to transmit effectively among insect generations and across lines and species can make them critical agents of rapid response and adaptation to environmental and biotic challenges and opportunities. These diverse effects can be reflected across insects' critical traits: abilities to exploit novel food sources, resist natural enemies, or vector diseases of other animals or plants. They can help insects resist various pressures of the Anthropocene era, including heat, drought, invasive species, and pollution with toxic chemicals.

Understanding insect microbiota is essential from both basic and applied perspectives. Characterizing and monitoring microbiota will help us understand changing natural ecosystems during times of global biodiversity crisis and may offer novel opportunities for species conservation. It will be key for managing insects in agricultural systems as we try to feed the growing global human population under changing environmental conditions, while reducing the applications of toxic chemicals and other negative environmental impacts. It will also help control diseases vectored by insects—those directly affecting humans, like dengue and related viruses, and those attacking other animals and plants that matter to ecosystem functions and the human economy.

Considering the microbial symbionts importance, the opportunities for insect microbiota characterization provided by increasing biodiversity surveys seem like a massive opportunity. By mining the growing number of insect biodiversity datasets, we could obtain information on some key microbial players in insect biology. High-throughput barcoding datasets and collections could serve as an ideal starting point for characterizing microbial transmission across interacting species that form natural communities. Including additional DNA-based tools in a step-wise manner could provide both a broad perspective and deep insights into the most promising insect–microbe associations. Implementing such routines into insect biodiversity monitoring attempts will provide valuable information about the basic biology of the planet's most diverse animal clade—and much-need insight into how not only insects but also their essential microbiomes can be effectively monitored, managed and protected in our rapidly changing world.

## Data Availability

This article has no additional data.

## References

[1] McGavin G. 1992 Insects of the northern hemisphere. Limpsfield, UK: Dragon's World.

[2] Wagner DL, Grames EM, Forister ML, Berenbaum MR, Stopak D. 2021 Insect decline in the Anthropocene: death by a thousand cuts. Proc. Natl Acad. Sci. USA **118**, e2023989118. (10.1073/pnas.2023989118) 33431573 PMC7812858

[3] McFall-Ngai M et al. 2013 Animals in a bacterial world, a new imperative for the life sciences. Proc. Natl Acad. Sci. USA **110**, 3229-3236. (10.1073/pnas.1218525110) 23391737 PMC3587249

[4] Sudakaran S, Kost C, Kaltenpoth M. 2017 Symbiont acquisition and replacement as a source of ecological innovation. Trends Microbiol. **25**, 375-390. (10.1016/j.tim.2017.02.014) 28336178

[5] Oliver KM, Degnan PH, Burke GR, Moran NA. 2010 Facultative symbionts in aphids and the horizontal transfer of ecologically important traits. Annu. Rev. Entomol. **55**, 247-266. (10.1146/annurev-ento-112408-085305) 19728837

[6] Ferrari J, Vavre F. 2011 Bacterial symbionts in insects or the story of communities affecting communities. Phil. Trans. R. Soc. B **366**, 1389-1400. (10.1098/rstb.2010.0226) 21444313 PMC3081568

[7] Douglas AE. 2021 The symbiotic habit. Princeton, NJ: Princeton University Press.

[8] Berg G et al. 2020 Microbiome definition re-visited: old concepts and new challenges. Microbiome **8**, 103. (10.1186/s40168-020-00875-0) 32605663 PMC7329523

[9] Moran NA, McCutcheon JP, Nakabachi A. 2008 Genomics and evolution of heritable bacterial symbionts. Annu. Rev. Genet. **42**, 165-190. (10.1146/annurev.genet.41.110306.130119) 18983256

[10] Perreau J, Moran NA. 2022 Genetic innovations in animal–microbe symbioses. Nat. Rev. Genet. **23**, 23-39. (10.1038/s41576-021-00395-z) 34389828 PMC8832400

[11] Hammer TJ, Sanders JG, Fierer N. 2019 Not all animals need a microbiome. FEMS Microbiol. Lett. **366**, fnz117. (10.1093/femsle/fnz117) 31132110

[12] Bennett GM, Moran NA. 2013 Small, smaller, smallest: the origins and evolution of ancient dual symbioses in a phloem-feeding insect. Genome Biol. Evol. **5**, 1675-1688. (10.1093/gbe/evt118) 23918810 PMC3787670

[13] Salem H et al. 2017 Drastic genome reduction in an herbivore's pectinolytic symbiont. Cell **171**, 1520-1531.e13. (10.1016/j.cell.2017.10.029) 29153832

[14] Husnik F, McCutcheon JP. 2016 Repeated replacement of an intrabacterial symbiont in the tripartite nested mealybug symbiosis. Proc. Natl Acad. Sci. USA **113**, E5416-E5424. (10.1073/pnas.1603910113) 27573819 PMC5027413

[15] Itoh H, Tago K, Hayatsu M, Kikuchi Y. 2018 Detoxifying symbiosis: microbe-mediated detoxification of phytotoxins and pesticides in insects. Nat. Prod. Rep. **35**, 434-454. (10.1039/C7NP00051K) 29644346

[16] Brownlie JC, Johnson KN. 2009 Symbiont-mediated protection in insect hosts. Trends Microbiol. **17**, 348-354. (10.1016/j.tim.2009.05.005) 19660955

[17] Gerardo NM, Parker BJ. 2014 Mechanisms of symbiont-conferred protection against natural enemies: an ecological and evolutionary framework. Curr. Opin. Insect Sci. **4**, 8-14. (10.1016/j.cois.2014.08.002) 28043411

[18] Vorburger C, Perlman SJ. 2018 The role of defensive symbionts in host–parasite coevolution. Biol. Rev. **93**, 1747-1764. (10.1111/brv.12417) 29663622

[19] Werren JH, Baldo L, Clark ME. 2008 *Wolbachia:* master manipulators of invertebrate biology. Nat. Rev. Microbiol. **6**, 741-751. (10.1038/nrmicro1969) 18794912

[20] Vorburger C. 2022 Defensive symbionts and the evolution of parasitoid host specialization. Annu. Rev. Entomol. **67**, 329-346. (10.1146/annurev-ento-072621-062042) 34614366

[21] Kiefer JST, Bauer E, Okude G, Fukatsu T, Kaltenpoth M, Engl T. 2023 Cuticle supplementation and nitrogen recycling by a dual bacterial symbiosis in a family of xylophagous beetles. ISME J. **17**, 1029-1039. (10.1038/s41396-023-01415-y) 37085551 PMC10284843

[22] Engel P, Moran NA. 2013 The gut microbiota of insects—diversity in structure and function. FEMS Microbiol. Rev. **37**, 699-735. (10.1111/1574-6976.12025) 23692388

[23] Drew GC, Stevens EJ, King KC. 2021 Microbial evolution and transitions along the parasite–mutualist continuum. Nat. Rev. Microbiol. **19**, 623-638. (10.1038/s41579-021-00550-7) 33875863 PMC8054256

[24] Clayton AL, Oakeson KF, Gutin M, Pontes A, Dunn DM, von Niederhausern AC, Weiss RB, Fisher M, Dale C. 2012 A novel human-infection-derived bacterium provides insights into the evolutionary origins of mutualistic insect–bacterial symbioses. PLoS Genet. **8**, e1002990. (10.1371/journal.pgen.1002990) 23166503 PMC3499248

[25] Matsuura Y, Moriyama M, Łukasik P, Vanderpool D, Tanahashi M, Meng X-Y, McCutcheon JP, Fukatsu T. 2018 Recurrent symbiont recruitment from fungal parasites in cicadas. Proc. Natl Acad. Sci. USA **115**, E5970-E5979. (10.1073/pnas.1803245115) 29891654 PMC6042066

[26] McCutcheon JP, Boyd BM, Dale C. 2019 The life of an insect endosymbiont from the cradle to the grave. Curr. Biol. **29**, R485-R495. (10.1016/j.cub.2019.03.032) 31163163

[27] Baumann P. 2005 Biology bacteriocyte-associated endosymbionts of plant sap-sucking insects. Annu. Rev. Microbiol. **59**, 155-189. (10.1146/annurev.micro.59.030804.121041) 16153167

[28] Husnik F. 2018 Host–symbiont–pathogen interactions in blood-feeding parasites: nutrition, immune cross-talk and gene exchange. Parasitology **145**, 1294-1303. (10.1017/S0031182018000574) 29642965

[29] Bennett GM, Moran NA. 2015 Heritable symbiosis: the advantages and perils of an evolutionary rabbit hole. Proc. Natl Acad. Sci. USA **112**, 10 169-10 176. (10.1073/pnas.1421388112) 25713367 PMC4547261

[30] Salem H, Bauer E, Strauss AS, Vogel H, Marz M, Kaltenpoth M. 2014 Vitamin supplementation by gut symbionts ensures metabolic homeostasis in an insect host. Proc. R. Soc. B **281**, 20141838. (10.1098/rspb.2014.1838) PMC4213650 25339726

[31] Kwong WK, Moran NA. 2016 Gut microbial communities of social bees. Nat. Rev. Microbiol. **14**, 374-384. (10.1038/nrmicro.2016.43) 27140688 PMC5648345

[32] Hu Y et al. 2018 Herbivorous turtle ants obtain essential nutrients from a conserved nitrogen-recycling gut microbiome. Nat. Commun. **9**, 1-14. (10.1038/s41467-018-03357-y) 29511180 PMC5840417

[33] Arora J et al. 2022 The functional evolution of termite gut microbiota. Microbiome **10**, 78. (10.1186/s40168-022-01258-3) 35624491 PMC9137090

[34] Dearing MD, Kaltenpoth M, Gershenzon J. 2022 Demonstrating the role of symbionts in mediating detoxification in herbivores. Symbiosis **87**, 59-66. (10.1007/s13199-022-00863-y) 36164313 PMC9499882

[35] Johnson AJ, McKenna DD, Jordal BH, Cognato AI, Smith SM, Lemmon AR, Lemmon EM, Hulcr J. 2018 Phylogenomics clarifies repeated evolutionary origins of inbreeding and fungus farming in bark beetles (Curculionidae, Scolytinae). Mol. Phylogenet. Evol. **127**, 229-238. (10.1016/j.ympev.2018.05.028) 29860101

[36] Mueller UG, Kardish MR, Ishak HD, Wright AM, Solomon SE, Bruschi SM, Carlson AL, Bacci Jr M. 2018 Phylogenetic patterns of ant–fungus associations indicate that farming strategies, not only a superior fungal cultivar, explain the ecological success of leafcutter ants. Mol. Ecol. **27**, 2414-2434. (10.1111/mec.14588) 29740906

[37] Vanderpool D, Bracewell RR, McCutcheon JP. 2018 Know your farmer: ancient origins and multiple independent domestications of ambrosia beetle fungal cultivars. Mol. Ecol. **27**, 2077-2094. (10.1111/mec.14394) 29087025

[38] Chouvenc T, Šobotník J, Engel MS, Bourguignon T. 2021 Termite evolution: mutualistic associations, key innovations, and the rise of Termitidae. Cell. Mol. Life Sci. **78**, 2749-2769. (10.1007/s00018-020-03728-z) 33388854 PMC11071720

[39] Hosokawa T, Imanishi M, Koga R, Fukatsu T. 2019 Diversity and evolution of bacterial symbionts in the gut symbiotic organ of jewel stinkbugs (Hemiptera: Scutelleridae). Appl. Entomol. Zool. **54**, 359-367. (10.1007/s13355-019-00630-4)

[40] Koga R et al. 2022 Single mutation makes *Escherichia coli* an insect mutualist. Nat. Microbiol. **7**, 1141-1150. (10.1038/s41564-022-01179-9) 35927448 PMC9352592

[41] Takeshita K, Kikuchi Y. 2020 Genomic comparison of insect gut symbionts from divergent *Burkholderia* subclades. Genes **11**, 744. (10.3390/genes11070744) 32635398 PMC7397029

[42] Hunter MS, Umanzor EF, Kelly SE, Whitaker SM, Ravenscraft A. 2022 Development of common leaf-footed bug pests depends on the presence and identity of their environmentally acquired symbionts. Appl. Environ. Microbiol. **88**, e01778-21. (10.1128/aem.01778-21) 34986009 PMC8904059

[43] Wang Y, Rozen DE. 2018 Gut microbiota in the burying beetle, *Nicrophorus vespilloides*, provide colonization resistance against larval bacterial pathogens. Ecol. Evol. **8**, 1646-1654. (10.1002/ece3.3589) 29435240 PMC5792511

[44] Shukla SP, Plata C, Reichelt M, Steiger S, Heckel DG, Kaltenpoth M, Vilcinskas A, Vogel H. 2018 Microbiome-assisted carrion preservation aids larval development in a burying beetle. Proc. Natl Acad. Sci. USA **115**, 11 274-11 279. (10.1073/pnas.1812808115) PMC6217399 30322931

[45] Engl T, Kroiss J, Kai M, Nechitaylo TY, Svatoš A, Kaltenpoth M. 2018 Evolutionary stability of antibiotic protection in a defensive symbiosis. Proc. Natl Acad. Sci. USA **115**, E2020-E2029. (10.1073/pnas.1719797115) 29444867 PMC5834716

[46] Kaltenpoth M, Göttler W, Herzner G, Strohm E. 2005 Symbiotic bacteria protect wasp larvae from fungal infestation. Curr. Biol. **15**, 475-479. (10.1016/j.cub.2004.12.084) 15753044

[47] Holmes NA et al*.* 2016 Genome analysis of two *Pseudonocardia* phylotypes associated with *Acromyrmex* leafcutter ants reveals their biosynthetic potential. Front. Microbiol. **7**, 235901. (10.3389/fmicb.2016.02073) PMC5183585 28082956

[48] Nakabachi A, Piel J, Malenovský I, Hirose Y. 2020 Comparative genomics underlines multiple roles of *Profftella*, an obligate symbiont of psyllids: providing toxins, vitamins, and carotenoids. Genome Biol. Evol. **12**, 1975-1987. (10.1093/gbe/evaa175) 32797185 PMC7643613

[49] Kremer N, Charif D, Henri H, Bataille M, Prévost G, Kraaijeveld K, Vavre F. 2009 A new case of *Wolbachia* dependence in the genus *Asobara*: evidence for parthenogenesis induction in *Asobara japonica*. Heredity **103**, 248-256. (10.1038/hdy.2009.63) 19513092

[50] Sanders JG, Łukasik P, Frederickson ME, Russell JA, Koga R, Knight R, Pierce NE. 2017 Dramatic differences in gut bacterial densities correlate with diet and habitat in rainforest ants. Integr. Comp. Biol. **57**, 705-722. (10.1093/icb/icx088) 28985400

[51] Lemoine MM, Engl T, Kaltenpoth M. 2020 Microbial symbionts expanding or constraining abiotic niche space in insects. Curr. Opin. Insect Sci. **39**, 14-20. (10.1016/j.cois.2020.01.003) 32086000

[52] Hosokawa T, Kikuchi Y, Shimada M, Fukatsu T. 2007 Obligate symbiont involved in pest status of host insect. Proc. R. Soc. B **274**, 1979-1984. (10.1098/rspb.2007.0620) PMC2275188 17567556

[53] Wagner SM, Martinez AJ, Ruan Y-M, Kim KL, Lenhart PA, Dehnel AC, Oliver KM, White JA. 2015 Facultative endosymbionts mediate dietary breadth in a polyphagous herbivore. Funct. Ecol. **29**, 1402-1410. (10.1111/1365-2435.12459)

[54] Chen B, Mason CJ, Peiffer M, Zhang D, Shao Y, Felton GW. 2022 Enterococcal symbionts of caterpillars facilitate the utilization of a suboptimal diet. J. Insect. Physiol. **138**, 104369. (10.1016/j.jinsphys.2022.104369) 35157920

[55] Chung SH, Rosa C, Scully ED, Peiffer M, Tooker JF, Hoover K, Luthe DS, Felton GW. 2013 Herbivore exploits orally secreted bacteria to suppress plant defenses. Proc. Natl Acad. Sci. USA **110**, 15 728-15 733. (10.1073/pnas.1308867110) PMC3785742 24019469

[56] Parish AJ, Rice DW, Tanquary VM, Tennessen JM, Newton ILG. 2022 Honey bee symbiont buffers larvae against nutritional stress and supplements lysine. ISME J. **16**, 2160-2168. (10.1038/s41396-022-01268-x) 35726020 PMC9381588

[57] Storelli G, Strigini M, Grenier T, Bozonnet L, Schwarzer M, Daniel C, Matos R, Leulier F. 2018 *Drosophila perpetuates* nutritional mutualism by promoting the fitness of its intestinal symbiont *Lactobacillus plantarum*. Cell Metab. **27**, 362-377.e8. (10.1016/j.cmet.2017.11.011) 29290388 PMC5807057

[58] Janke RS, Kaftan F, Niehs SP, Scherlach K, Rodrigues A, Svatoš A, Hertweck C, Kaltenpoth M, Flórez LV. 2022 Bacterial ectosymbionts in cuticular organs chemically protect a beetle during molting stages. ISME J. **16**, 2691-2701. (10.1038/s41396-022-01311-x) 36056153 PMC9666510

[59] Costopoulos K, Kovacs JL, Kamins A, Gerardo NM. 2014 Aphid facultative symbionts reduce survival of the predatory lady beetle *Hippodamia convergens**.* BMC Ecol. **14**, 5. (10.1186/1472-6785-14-5) 24555501 PMC3936903

[60] Kovacs JL, Wolf C, Voisin D, Wolf S. 2017 Evidence of indirect symbiont conferred protection against the predatory lady beetle *Harmonia axyridis* in the pea aphid. BMC Ecol. **17**, 26. (10.1186/s12898-017-0136-x) 28693550 PMC5504669

[61] Łukasik P, van Asch M, Guo H, Ferrari J, Godfray HCJ. 2013 Unrelated facultative endosymbionts protect aphids against a fungal pathogen. Ecol. Lett. **16**, 214-218. (10.1111/ele.12031) 23137173

[62] Zhao D, Zhang Z, Niu H, Guo H. 2023 Pathogens are an important driving force for the rapid spread of symbionts in an insect host. Nat. Ecol. Evol. **7**, 1667-1681. (10.1038/s41559-023-02160-3) 37563464

[63] Oliver KM, Moran NA, Hunter MS. 2005 Variation in resistance to parasitism in aphids is due to symbionts not host genotype. Proc. Natl Acad. Sci. USA **102**, 12 795-12 800. (10.1073/pnas.0506131102) PMC1200300 16120675

[64] Xie J, Vilchez I, Mateos M. 2010 *Spiroplasma* bacteria enhance survival of *Drosophila hydei* attacked by the parasitic wasp *Leptopilina heterotoma*. PLoS ONE **5**, e12149. (10.1371/journal.pone.0012149) 20730104 PMC2921349

[65] Cockburn SN, Haselkorn TS, Hamilton PT, Landzberg E, Jaenike J, Perlman SJ. 2013 Dynamics of the continent-wide spread of a *Drosophila* defensive symbiont. Ecol. Lett. **16**, 609-616. (10.1111/ele.12087) 23517577

[66] Jaenike J, Unckless R, Cockburn SN, Boelio LM, Perlman SJ. 2010 Adaptation via symbiosis: recent spread of a *Drosophila* defensive symbiont. Science **329**, 212-215. (10.1126/science.1188235) 20616278

[67] Hajek AE, Morris EE, Hendry TA. 2019 Context-dependent interactions of insects and defensive symbionts: insights from a novel system in siricid woodwasps. Curr. Opin. Insect Sci. **33**, 77-83. (10.1016/j.cois.2019.03.006) 31358200

[68] Skowronek M, Sajnaga E, Pleszczyńska M, Kazimierczak W, Lis M, Wiater A. 2020 Bacteria from the midgut of common cockchafer (*Melolontha melolontha* L.) larvae exhibiting antagonistic activity against bacterial symbionts of entomopathogenic nematodes: isolation and molecular identification. Int. J. Mol. Sci. **21**, 580. (10.3390/ijms21020580) 31963214 PMC7013910

[69] Koch H, Schmid-Hempel P. 2011 Socially transmitted gut microbiota protect bumble bees against an intestinal parasite. Proc. Natl Acad. Sci. USA **108**, 19 288-19 292. (10.1073/pnas.1110474108) PMC3228419 22084077

[70] Yoshiyama M, Kimura K. 2009 Bacteria in the gut of Japanese honeybee, *Apis cerana japonica*, and their antagonistic effect against *Paenibacillus larvae*, the causal agent of American foulbrood. J. Invertebr. Pathol. **102**, 91-96. (10.1016/j.jip.2009.07.005) 19616552

[71] Martinez J, Tolosana I, Ok S, Smith S, Snoeck K, Day JP, Jiggins FM. 2017 Symbiont strain is the main determinant of variation in *Wolbachia*-mediated protection against viruses across *Drosophila* species. Mol. Ecol. **26**, 4072-4084. (10.1111/mec.14164) 28464440 PMC5966720

[72] Moreira LA et al. 2009 A *Wolbachia* symbiont in *Aedes aegypti* limits infection with dengue, chikungunya, and *Plasmodium*. Cell **139**, 1268-1278. (10.1016/j.cell.2009.11.042) 20064373

[73] Gong J-T et al. 2020 Stable introduction of plant-virus-inhibiting *Wolbachia* into planthoppers for rice protection. Curr. Biol. **30**, 4837-4845.e5. (10.1016/j.cub.2020.09.033) 33035486

[74] Heyworth ER, Smee MR, Ferrari J. 2020 Aphid facultative symbionts aid recovery of their obligate symbiont and their host after heat stress. Front. Ecol. Evol. **8**, 56. (10.3389/fevo.2020.00056)

[75] Tougeron K, Iltis C, Rampnoux E, Goerlinger A, Dhondt L, Hance T. 2023 Still standing: the heat protection delivered by a facultative symbiont to its aphid host is resilient to repeated thermal stress. Curr. Res. Insect Sci. **3**, 100061. (10.1016/j.cris.2023.100061) 37304568 PMC10250925

[76] Burdina EV, Bykov RA, Menshanov PN, Ilinsky YY, Gruntenko NЕ. 2021 Unique *Wolbachia* strain wMelPlus increases heat stress resistance in *Drosophila melanogaster**.* Arch. Insect Biochem. Physiol. **106**, e21776. (10.1002/arch.21776) 33644932

[77] Kikuchi Y, Hayatsu M, Hosokawa T, Nagayama A, Tago K, Fukatsu T. 2012 Symbiont-mediated insecticide resistance. Proc. Natl Acad. Sci. USA **109**, 8618-8622. (10.1073/pnas.1200231109) 22529384 PMC3365206

[78] Senderovich Y, Halpern M. 2013 The protective role of endogenous bacterial communities in chironomid egg masses and larvae. ISME J. **7**, 2147-2158. (10.1038/ismej.2013.100) 23804150 PMC3806255

[79] Ceja-Navarro JA et al. 2015 Gut microbiota mediate caffeine detoxification in the primary insect pest of coffee. Nat. Commun. **6**, 7618. (10.1038/ncomms8618) 26173063 PMC4510693

[80] Cass BN, Himler AG, Bondy EC, Bergen JE, Fung SK, Kelly SE, Hunter MS. 2016 Conditional fitness benefits of the *Rickettsia* bacterial symbiont in an insect pest. Oecologia **180**, 169-179. (10.1007/s00442-015-3436-x) 26376661

[81] Weeks AR, Turelli M, Harcombe WR, Reynolds KT, Hoffmann AA. 2007 From parasite to mutualist: rapid evolution of *Wolbachia* in natural populations of *Drosophila*. PLoS Biol. **5**, e114. (10.1371/journal.pbio.0050114) 17439303 PMC1852586

[82] Kriesner P, Hoffmann AA, Lee SF, Turelli M, Weeks AR. 2013 Rapid sequential spread of two *Wolbachia* variants in *Drosophila simulans*. PLoS Pathog. **9**, e1003607. (10.1371/journal.ppat.1003607) 24068927 PMC3771877

[83] Hurst GDD, Frost CL. 2015 Reproductive parasitism: maternally inherited symbionts in a biparental world. Cold Spring Harb. Perspect. Biol. **7**, a017699. (10.1101/cshperspect.a017699) 25934011 PMC4448626

[84] Hornett EA, Kageyama D, Hurst GDD. 2022 Sex determination systems as the interface between male-killing bacteria and their hosts. Proc. R. Soc. B **289**, 20212781. (10.1098/rspb.2021.2781) PMC9005997 35414231

[85] Leonardo TE, Mondor EB. 2006 Symbiont modifies host life-history traits that affect gene flow. Proc. R. Soc. B **273**, 1079-1084. (10.1098/rspb.2005.3408) PMC1560252 16600884

[86] Tsuchida T, Koga R, Fukatsu T. 2004 Host plant specialization governed by facultative symbiont. Science **303**, 1989. (10.1126/science.1094611) 15044797

[87] Ferrari J, Scarborough CL, Godfray HCJ. 2007 Genetic variation in the effect of a facultative symbiont on host-plant use by pea aphids. Oecologia **153**, 323-329. (10.1007/s00442-007-0730-2) 17415589

[88] Brownlie JC, Cass BN, Riegler M, Witsenburg JJ, Iturbe-Ormaetxe I, McGraw EA, O'Neill SL. 2009 Evidence for metabolic provisioning by a common invertebrate endosymbiont, *Wolbachia pipientis*, during periods of nutritional stress. PLoS Pathog. **5**, e1000368. (10.1371/journal.ppat.1000368) 19343208 PMC2657209

[89] Frago E, Mala M, Weldegergis BT, Yang C, McLean A, Godfray HCJ, Gols R, Dicke M. 2017 Symbionts protect aphids from parasitic wasps by attenuating herbivore-induced plant volatiles. Nat. Commun. **8**, 1860. (10.1038/s41467-017-01935-0) 29192219 PMC5709398

[90] Łukasik P, Dawid MA, Ferrari J, Godfray HCJ. 2013 The diversity and fitness effects of infection with facultative endosymbionts in the grain aphid, *Sitobion avenae*. Oecologia **173**, 985-996. (10.1007/s00442-013-2660-5) 23624672

[91] José de Souza D, Devers S, Lenoir A. 2011 *Blochmannia* endosymbionts and their host, the ant *Camponotus fellah*: cuticular hydrocarbons and melanization. C. R. Biol. **334**, 737-741. (10.1016/j.crvi.2011.06.008) 21943523

[92] Kanyile SN, Engl T, Heddi A, Kaltenpoth M. 2023 Endosymbiosis allows S*itophilus oryzae* to persist in dry conditions. Front. Microbiol. **14**, 1199370. (10.3389/fmicb.2023.1199370) 37497544 PMC10366622

[93] Dion E, Polin SE, Simon J-C, Outreman Y. 2011 Symbiont infection affects aphid defensive behaviours. Biol. Lett. **7**, 743-746. (10.1098/rsbl.2011.0249) 21490007 PMC3169066

[94] Brumin M, Kontsedalov S, Ghanim M. 2011 *Rickettsia* influences thermotolerance in the whitefly *Bemisia tabaci* B biotype. Insect Sci. **18**, 57-66. (10.1111/j.1744-7917.2010.01396.x)

[95] Corbin C, Heyworth ER, Ferrari J, Hurst GDD. 2017 Heritable symbionts in a world of varying temperature. Heredity **118**, 10-20. (10.1038/hdy.2016.71) 27703153 PMC5176117

[96] Rothman JA, Leger L, Graystock P, Russell K, McFrederick QS. 2019 The bumble bee microbiome increases survival of bees exposed to selenate toxicity. Environ. Microbiol. **21**, 3417-3429. (10.1111/1462-2920.14641) 31026366

[97] Kikuchi Y, Tada A, Musolin DL, Hari N, Hosokawa T, Fujisaki K, Fukatsu T. 2016 Collapse of insect gut symbiosis under simulated climate change. mBio **7**, 10.1128/mbio.01578-16. (10.1128/mbio.01578-16) PMC5050343 27703075

[98] Dunbar HE, Wilson ACC, Ferguson NR, Moran NA. 2007 Aphid thermal tolerance Is governed by a point mutation in bacterial symbionts. PLoS Biol. **5**, e96. (10.1371/journal.pbio.0050096) 17425405 PMC1847839

[99] Harmon JP, Moran NA, Ives AR. 2009 Species response to environmental change: impacts of food web interactions and evolution. Science **323**, 1347-1350. (10.1126/science.1167396) 19265021

[100] Motta EVS, Raymann K, Moran NA. 2018 Glyphosate perturbs the gut microbiota of honey bees. Proc. Natl Acad. Sci. USA **115**, 10 305-10 310. (10.1073/pnas.1803880115) PMC6187125 30249635

[101] Simon J-C, Boutin S, Tsuchida T, Koga R, Gallic J-FL, Frantz A, Outreman Y, Fukatsu T. 2011 Facultative symbiont infections affect aphid reproduction. PLoS ONE **6**, e21831. (10.1371/journal.pone.0021831) 21818272 PMC3144876

[102] Zytynska SE, Tighiouart K, Frago E. 2021 Benefits and costs of hosting facultative symbionts in plant-sucking insects: a meta-analysis. Mol. Ecol. **30**, 2483-2494. (10.1111/mec.15897) 33756029

[103] Braendle C, Davis GK, Brisson JA, Stern DL. 2006 Wing dimorphism in aphids. Heredity **97**, 192-199. (10.1038/sj.hdy.6800863) 16823401

[104] Lopez V, Cortesero AM, Poinsot D. 2018 Influence of the symbiont *Wolbachia* on life history traits of the cabbage root fly (*Delia radicum*). J. Invertebr. Pathol. **158**, 24-31. (10.1016/j.jip.2018.09.002) 30193778

[105] Rothacher L, Ferrer-Suay M, Vorburger C. 2016 Bacterial endosymbionts protect aphids in the field and alter parasitoid community composition. Ecology **97**, 1712-1723. (10.1890/15-2022.1) 27859175

[106] Hrček J, McLean AHC, Godfray HCJ. 2016 Symbionts modify interactions between insects and natural enemies in the field. J. Anim. Ecol. **85**, 1605-1612. (10.1111/1365-2656.12586) 27561159 PMC5082498

[107] Łukasik P, Guo H, van Asch M, Ferrari J, Godfray HCJ. 2013 Protection against a fungal pathogen conferred by the aphid facultative endosymbionts *Rickettsia* and *Spiroplasma* is expressed in multiple host genotypes and species and is not influenced by co-infection with another symbiont. J. Evol. Biol. **26**, 2654-2661. (10.1111/jeb.12260) 24118386

[108] Haselkorn TS, Jaenike J. 2015 Macroevolutionary persistence of heritable endosymbionts: acquisition, retention and expression of adaptive phenotypes in *Spiroplasma*. Mol. Ecol. **24**, 3752-3765. (10.1111/mec.13261) 26053523

[109] Hoffmann AA et al. 2011 Successful establishment of *Wolbachia* in *Aedes* populations to suppress dengue transmission. Nature **476**, 454-457. (10.1038/nature10356) 21866160

[110] Rouchet R, Vorburger C. 2014 Experimental evolution of parasitoid infectivity on symbiont-protected hosts leads to the emergence of genotype specificity. Evolution **68**, 1607-1616. (10.1111/evo.12377) 24495148

[111] Wu T, Monnin D, Lee RAR, Henry LM. 2022 Local adaptation to hosts and parasitoids shape *Hamiltonella defensa* genotypes across aphid species. Proc. R. Soc. B **289**, 20221269. (10.1098/rspb.2022.1269) PMC9597410 36285493

[112] Kageyama D, Anbutsu H, Shimada M, Fukatsu T. 2009 Effects of host genotype against the expression of *Spiroplasma*-induced male killing in *Drosophila melanogaster**.* Heredity **102**, 475-482. (10.1038/hdy.2009.14) 19223920

[113] Sanaei E, Charlat S, Engelstädter J. 2021 *Wolbachia* host shifts: routes, mechanisms, constraints and evolutionary consequences. Biol. Rev. **96**, 433-453. (10.1111/brv.12663) 33128345

[114] Bensadia F, Boudreault S, Guay J-F, Michaud D, Cloutier C. 2006 Aphid clonal resistance to a parasitoid fails under heat stress. J. Insect Physiol. **52**, 146-157. (10.1016/j.jinsphys.2005.09.011) 16307754

[115] Chrostek E, Martins N, Marialva MS, Teixeira L. 2021 *Wolbachia*-conferred antiviral protection is determined by developmental temperature. mBio **12**, 10.1128/mbio.02923-20. (10.1128/mbio.02923-20) PMC8546536 34488458

[116] Hague MTJ, Shropshire JD, Caldwell CN, Statz JP, Stanek KA, Conner WR, Cooper BS. 2022 Temperature effects on cellular host–microbe interactions explain continent-wide endosymbiont prevalence. Curr. Biol. **32**, 878-888.e8. (10.1016/j.cub.2021.11.065) 34919808 PMC8891084

[117] Weldon SR, Russell JA, Oliver KM. 2019 More is not always better: coinfections with defensive symbionts generate highly variable outcomes. Appl. Environ. Microbiol. **86**, e02537-19. (10.1128/AEM.02537-19) PMC7028961 31862723

[118] Rock DI, Smith AH, Joffe J, Albertus A, Wong N, O'Connor M, Oliver KM, Russell JA. 2018 Context-dependent vertical transmission shapes strong endosymbiont community structure in the pea aphid, *Acyrthosiphon pisum*. Mol. Ecol. **27**, 2039-2056. (10.1111/mec.14449) 29215202

[119] Kaur R, Shropshire JD, Cross KL, Leigh B, Mansueto AJ, Stewart V, Bordenstein SR, Bordenstein SR. 2021 Living in the endosymbiotic world of *Wolbachia:* a centennial review. Cell Host Microbe **29**, 879-893. (10.1016/j.chom.2021.03.006) 33945798 PMC8192442

[120] Weinert LA, Araujo-Jnr EV, Ahmed MZ, Welch JJ. 2015 The incidence of bacterial endosymbionts in terrestrial arthropods. Proc. R. Soc. B **282**, 20150249. (10.1098/rspb.2015.0249) PMC4424649 25904667

[121] Zug R, Hammerstein P. 2015 Bad guys turned nice? A critical assessment of *Wolbachia* mutualisms in arthropod hosts. Biol. Rev. **90**, 89-111. (10.1111/brv.12098) 24618033

[122] Dedeine F, Boulétreau M, Vavre F. 2005 *Wolbachia* requirement for oogenesis: occurrence within the genus *Asobara* (Hymenoptera, Braconidae) and evidence for intraspecific variation in *A. tabida*. Heredity **95**, 394-400. (10.1038/sj.hdy.6800739) 16118660

[123] Hedges LM, Brownlie JC, O'Neill SL, Johnson KN. 2008 *Wolbachia* and virus protection in insects. Science **322**, 702. (10.1126/science.1162418) 18974344

[124] Higashi CHV, Kamalaker B, Patel V, Inaganti R, Bressan A, Russell JA, Oliver KM. 2023 ANOTHER TOOL IN THE TOOLBOX: *Wolbachia*-mediated protection against a specialized fungal pathogen of aphids. *bioRxiv* 2023.07.24.550390. (10.1101/2023.07.24.550390) 39562330

[125] Nikoh N, Hosokawa T, Moriyama M, Oshima K, Hattori M, Fukatsu T. 2014 Evolutionary origin of insect–*Wolbachia* nutritional mutualism. Proc. Natl Acad. Sci. USA **111**, 10 257-10 262. (10.1073/pnas.1409284111) PMC4104916 24982177

[126] Turelli M et al. 2018 Rapid global spread of wRi-like *Wolbachia* across multiple *Drosophila*. Curr. Biol. **28**, 963-971.e8. (10.1016/j.cub.2018.02.015) 29526588 PMC5882237

[127] Vancaester E, Blaxter M. 2023 Phylogenomic analysis of *Wolbachia* genomes from the Darwin Tree of Life biodiversity genomics project. PLoS Biol. **21**, e3001972. (10.1371/journal.pbio.3001972) 36689552 PMC9894559

[128] Tolley SJA, Nonacs P, Sapountzis P. 2019 *Wolbachia* horizontal transmission events in ants: what do we know and what can we learn? Front. Microbiol. **10**, 429888. (10.3389/fmicb.2019.00296) PMC6414450 30894837

[129] Jaenike J. 2012 Population genetics of beneficial heritable symbionts. Trends Ecol. Evol. **27**, 226-232. (10.1016/j.tree.2011.10.005) 22104387

[130] Tsuchida T, Koga R, Shibao H, Matsumoto T, Fukatsu T. 2002 Diversity and geographic distribution of secondary endosymbiotic bacteria in natural populations of the pea aphid, *Acyrthosiphon pisum*. Mol. Ecol. **11**, 2123-2135. (10.1046/j.1365-294X.2002.01606.x) 12296954

[131] Oliver KM, Smith AH, Russell JA. 2014 Defensive symbiosis in the real world—advancing ecological studies of heritable, protective bacteria in aphids and beyond. Funct. Ecol. **28**, 341-355. (10.1111/1365-2435.12133)

[132] Smith AH et al. 2015 Patterns, causes and consequences of defensive microbiome dynamics across multiple scales. Mol. Ecol. **24**, 1135-1149. (10.1111/mec.13095) 25683348

[133] Smith AH et al. 2021 Does getting defensive get you anywhere?—Seasonal balancing selection, temperature, and parasitoids shape real-world, protective endosymbiont dynamics in the pea aphid. Mol. Ecol. **30**, 2449-2472. (10.1111/mec.15906) 33876478

[134] Carpenter M, Peng L, Smith AH, Joffe J, O'Connor M, Oliver KM, Russell JA. 2021 Frequent drivers, occasional passengers: signals of symbiont-driven seasonal adaptation and hitchhiking in the pea aphid, *Acyrthosiphon pisum*. Insects **12**, 805. (10.3390/insects12090805) 34564245 PMC8466206

[135] Oliver KM, Campos J, Moran NA, Hunter MS. 2007 Population dynamics of defensive symbionts in aphids. Proc. R. Soc. B **275**, 293-299. (10.1098/rspb.2007.1192) PMC2593717 18029301

[136] Jaenike J, Brekke TD. 2011 Defensive endosymbionts: a cryptic trophic level in community ecology. Ecol. Lett. **14**, 150-155. (10.1111/j.1461-0248.2010.01564.x) 21155960

[137] Yadav S, Frazer J, Banga A, Pruitt K, Harsh S, Jaenike J, Eleftherianos I. 2018 Endosymbiont-based immunity in *Drosophila melanogaster* against parasitic nematode infection. PLoS ONE **13**, e0192183. (10.1371/journal.pone.0192183) 29466376 PMC5821453

[138] Himler AG et al. 2011 Rapid spread of a bacterial symbiont in an invasive whitefly is driven by fitness benefits and female bias. Science **332**, 254-256. (10.1126/science.1199410) 21474763

[139] Blanton AG, Peterson BF. 2020 Symbiont-mediated insecticide detoxification as an emerging problem in insect pests. Front. Microbiol. **11**, 547108. (10.3389/fmicb.2020.547108) 33101225 PMC7554331

[140] Raychoudhury R, Grillenberger BK, Gadau J, Bijlsma R, van de Zande L, Werren JH, Beukeboom LW. 2010 Phylogeography of *Nasonia vitripennis* (Hymenoptera) indicates a mitochondrial–*Wolbachia* sweep in North America. Heredity **104**, 318-326. (10.1038/hdy.2009.160) 20087396

[141] Deng J et al. 2021 *Wolbachia*-driven selective sweep in a range expanding insect species. BMC Ecol. Evol. **21**, 181. (10.1186/s12862-021-01906-6) 34563127 PMC8466699

[142] Carroll SP, Fox CW. 2008 Conservation biology: evolution in action. Oxford, UK: Oxford University Press.

[143] Miller WJ, Ehrman L, Schneider D. 2010 Infectious speciation revisited: impact of symbiont-depletion on female fitness and mating behavior of *Drosophila paulistorum*. PLoS Pathog. **6**, e1001214. (10.1371/journal.ppat.1001214) 21151959 PMC2996333

[144] Baião GC, Schneider DI, Miller WJ, Klasson L. 2019 The effect of *Wolbachia* on gene expression in *Drosophila paulistorum* and its implications for symbiont-induced host speciation. BMC Genomics **20**, 465. (10.1186/s12864-019-5816-9) 31174466 PMC6555960

[145] Husnik F, McCutcheon JP. 2018 Functional horizontal gene transfer from bacteria to eukaryotes. Nat. Rev. Microbiol. **16**, 67-79. (10.1038/nrmicro.2017.137) 29176581

[146] McLean AH, Godfray HCJ. 2015 Evidence for specificity in symbiont-conferred protection against parasitoids. Proc. R. Soc. B **282**, 20150977. (10.1098/rspb.2015.0977) PMC4528558 26136451

[147] Oliver KM, Higashi CH. 2019 Variations on a protective theme: *Hamiltonella defensa* infections in aphids variably impact parasitoid success. Curr. Opin. Insect Sci. **32**, 1-7. (10.1016/j.cois.2018.08.009) 31113620

[148] Casteel CL, Hansen AK, Walling LL, Paine TD. 2012 Manipulation of plant defense responses by the tomato psyllid (*Bactericerca cockerelli*) and its associated endosymbiont *Candidatus* Liberibacter psyllaurous. PLoS ONE **7**, e35191. (10.1371/journal.pone.0035191) 22539959 PMC3335145

[149] Heimpel GE, Abram PK, Brodeur J. 2021 A phylogenetic perspective on parasitoid host ranges with implications for biological control. Curr. Opin. Insect Sci. **44**, 95-100. (10.1016/j.cois.2021.04.003) 33901732

[150] Sanders D, Kehoe R, van Veen FF, McLean A, Godfray HCJ, Dicke M, Gols R, Frago E. 2016 Defensive insect symbiont leads to cascading extinctions and community collapse. Ecol. Lett. **19**, 789-799. (10.1111/ele.12616) 27282315 PMC4949664

[151] Frago E, Dicke M, Godfray HCJ. 2012 Insect symbionts as hidden players in insect–plant interactions. Trends Ecol. Evol. **27**, 705-711. (10.1016/j.tree.2012.08.013) 22985943

[152] Carroll AC, Wong A. 2018 Plasmid persistence: costs, benefits, and the plasmid paradox. Can. J. Microbiol. **64**, 293-304. (10.1139/cjm-2017-0609) 29562144

[153] Łukasik P, Guo H, van Asch M, Henry LM, Godfray HCJ, Ferrari J. 2015 Horizontal transfer of facultative endosymbionts is limited by host relatedness. Evolution **69**, 2757-2766. (10.1111/evo.12767) 26332792

[154] Henry LM, Peccoud J, Simon J-C, Hadfield JD, Maiden MJ, Ferrari J, Godfray HCJ. 2013 Horizontally transmitted symbionts and host colonization of ecological niches. Curr. Biol. **23**, 1713-1717. (10.1016/j.cub.2013.07.029) 23993843 PMC3980636

[155] Valdivia C, Newton JA, von Beeren C, O'Donnell S, Kronauer DJC, Russell JA, Łukasik P. 2023 Microbial symbionts are shared between ants and their associated beetles. Environ. Microbiol. **25**, 3466-3483. (10.1111/1462-2920.16544) 37968789

[156] Solomon S, Plattner G-K, Knutti R, Friedlingstein P. 2009 Irreversible climate change due to carbon dioxide emissions. Proc. Natl Acad. Sci. USA **106**, 1704-1709. (10.1073/pnas.0812721106) 19179281 PMC2632717

[157] Ritchie H, Roser M, Rosado P. 2022 Our World In Data. Retrieved from: https://ourworldindata.org/pesticides [Online Resource]

[158] Fletcher RJ et al. 2018 Is habitat fragmentation good for biodiversity? Biol. Conserv. **226**, 9-15. (10.1016/j.biocon.2018.07.022)

[159] Platts PJ, Mason SC, Palmer G, Hill JK, Oliver TH, Powney GD, Fox R, Thomas CD. 2019 Habitat availability explains variation in climate-driven range shifts across multiple taxonomic groups. Sci. Rep. **9**, 15039. (10.1038/s41598-019-51582-2) 31636341 PMC6803766

[160] Cronin JT, Haynes KJ. 2004 An invasive plant promotes unstable host–parasitoid patch dynamics. Ecology **85**, 2772-2782. (10.1890/04-0303)

[161] Sunny A, Diwakar S, Sharma GP. 2015 Native insects and invasive plants encounters. Arthropod Plant Interact. **9**, 323-331. (10.1007/s11829-015-9384-x)

[162] Cardoso P et al. 2020 Scientists' warning to humanity on insect extinctions. Biol. Conserv. **242**, 108426. (10.1016/j.biocon.2020.108426)

[163] McCulloch GA, Waters JM. 2023 Rapid adaptation in a fast-changing world: emerging insights from insect genomics. Glob. Change Biol. **29**, 943-954. (10.1111/gcb.16512) PMC10100130 36333958

[164] Bright M, Bulgheresi S. 2010 A complex journey: transmission of microbial symbionts. Nat. Rev. Microbiol. **8**, 218-230. (10.1038/nrmicro2262) 20157340 PMC2967712

[165] Bockoven AA, Bondy EC, Flores MJ, Kelly SE, Ravenscraft AM, Hunter MS. 2020 What goes up might come down: the spectacular spread of an endosymbiont is followed by its decline a decade later. Microb. Ecol. **79**, 482-494. (10.1007/s00248-019-01417-4) 31407021

[166] Knight R et al. 2018 Best practices for analysing microbiomes. Nat. Rev. Microbiol. **16**, 410-422. (10.1038/s41579-018-0029-9) 29795328

[167] Kolasa M, Kajtoch Ł, Michalik A, Maryańska-Nadachowska A, Łukasik P. 2023 Till evolution do us part: the diversity of symbiotic associations across populations of *Philaenus* spittlebugs. Environ. Microbiol. **25**, 2431-2446. (10.1111/1462-2920.16473) 37525959

[168] Chong RA, Moran NA. 2018 Evolutionary loss and replacement of *Buchnera*, the obligate endosymbiont of aphids. ISME J. **12**, 898-908. (10.1038/s41396-017-0024-6) 29362506 PMC5864228

[169] Mamanova L, Coffey AJ, Scott CE, Kozarewa I, Turner EH, Kumar A, Howard E, Shendure J, Turner DJ. 2010 Target-enrichment strategies for next-generation sequencing. Nat. Methods **7**, 111-118. (10.1038/nmeth.1419) 20111037

[170] Simon C et al. 2019 Off-target capture data, endosymbiont genes and morphology reveal a relict lineage that is sister to all other singing cicadas. Biol. J. Linn. Soc. **128**, 865-886. (10.1093/biolinnean/blz120)

[171] Dunning Hotopp JC, Slatko BE, Foster JM. 2017 Targeted enrichment and sequencing of recent endosymbiont-host lateral gene transfers. Sci. Rep. **7**, 857. (10.1038/s41598-017-00814-4) 28405008 PMC5429809

[172] Chua PYS, Bourlat SJ, Ferguson C, Korlevic P, Zhao L, Ekrem T, Meier R, Lawniczak MKN. 2023 Future of DNA-based insect monitoring. Trends Genet. **39**, 531-544. (10.1016/j.tig.2023.02.012) 36907721

[173] Iwaszkiewicz-Eggebrecht E et al. 2023 Optimizing insect metabarcoding using replicated mock communities. Methods Ecol. Evol. **14**, 1130-1146. (10.1111/2041-210X.14073) 37876735 PMC10593422

[174] Smith MA et al. 2012 *Wolbachia* and DNA barcoding insects: patterns, potential, and problems. PLoS ONE **7**, e36514. (10.1371/journal.pone.0036514) 22567162 PMC3342236

[175] Nowak K. 2022 Identifying patterns in microbiome composition across insect communities. Krakow, Poland: Jagiellonian University.

[176] Scholz M, Albanese D, Tuohy K, Donati C, Segata N, Rota-Stabelli O. 2020 Large scale genome reconstructions illuminate *Wolbachia* evolution. Nat. Commun. **11**, 5235. (10.1038/s41467-020-19016-0) 33067437 PMC7568565

[177] Hartop E, Srivathsan A, Ronquist F, Meier R. 2022 Towards Large-scale Integrative Taxonomy (LIT): resolving the data conundrum for dark taxa. Syst. Biol. **71**, 1404-1422. (10.1093/sysbio/syac033) 35556139 PMC9558837

[178] Srivathsan A et al. 2023 Convergence of dominance and neglect in flying insect diversity. Nat. Ecol. Evol. **7**, 1012-1021. (10.1038/s41559-023-02066-0) 37202502 PMC10333119

[179] Srivathsan A, Lee L, Katoh K, Hartop E, Kutty SN, Wong J, Yeo D, Meier R. 2021 ONTbarcoder and MinION barcodes aid biodiversity discovery and identification by everyone, for everyone BMC Biol. **19**, 217. (10.1186/s12915-021-01141-x) 34587965 PMC8479912

[180] Andriienko V. 2023 High-throughput insect barcoding in microbiome studies: impact of non-destructive DNA extraction on microbiome composition. Krakow: Jagiellonian University. See https://ruj.uj.edu.pl/xmlui/handle/item/312596. 10.7717/peerj.18025 PMC11426317 39329134

[181] Wetterstrand KA. 2023 *DNA sequencing costs: data from the NHGRI genome sequencing program (GSP)*. Bethesda, MD: National Human Genome Research Institute. See www.genome.gov/sequencingcostsdata.

[182] Wang H et al. 2023 Scientific discovery in the age of artificial intelligence. Nature **620**, 47-60. (10.1038/s41586-023-06221-2) 37532811

[183] Fink C, Staubach F, Kuenzel S, Baines JF, Roeder T. 2013 Noninvasive analysis of microbiome dynamics in the fruit fly *Drosophila melanogaster*. Appl. Environ. Microbiol. **79**, 6984-6988. (10.1128/AEM.01903-13) 24014528 PMC3811555

[184] Xu LT, Lou QZ, Cheng CH, Lu M, Sun JH. 2015 Gut-associated bacteria of *Dendroctonus valens* and their involvement in verbenone production. Microb. Ecol. **70**, 1012-1023. (10.1007/s00248-015-0625-4) 25985770

[185] Morrill A, Forbes MR, Vesterinen EJ, Tamminen M, Sääksjärvi IE, Kaunisto KM. 2023 Molecular characterisation of faecal bacterial assemblages among four species of syntopic odonates. Microb. Ecol. **87**, 16. (10.1007/s00248-023-02328-1) 38108886 PMC10728244

